# Mitochondrial genome evolution in Alismatales: Size reduction and extensive loss of ribosomal protein genes

**DOI:** 10.1371/journal.pone.0177606

**Published:** 2017-05-17

**Authors:** Gitte Petersen, Argelia Cuenca, Athanasios Zervas, Gregory T. Ross, Sean W. Graham, Craig F. Barrett, Jerrold I. Davis, Ole Seberg

**Affiliations:** 1 Natural History Museum of Denmark, University of Copenhagen, Copenhagen, Denmark; 2 Department of Botany, University of British Columbia, Vancouver, British Columbia, Canada; 3 UBC Botanical Garden & Centre for Plant Research, University of British Columbia, Vancouver, British Columbia, Canada; 4 L. H. Bailey Hortorium and Plant Biology Section, Cornell University, Ithaca, New York, United States of America; Ben-Gurion University of the Negev, ISRAEL

## Abstract

The order Alismatales is a hotspot for evolution of plant mitochondrial genomes characterized by remarkable differences in genome size, substitution rates, RNA editing, retrotranscription, gene loss and intron loss. Here we have sequenced the complete mitogenomes of *Zostera marina* and *Stratiotes aloides*, which together with previously sequenced mitogenomes from *Butomus* and *Spirodela*, provide new evolutionary evidence of genome size reduction, gene loss and transfer to the nucleus. The *Zostera* mitogenome includes a large portion of DNA transferred from the plastome, yet it is the smallest known mitogenome from a non-parasitic plant. Using a broad sample of the Alismatales, the evolutionary history of ribosomal protein gene loss is analyzed. In *Zostera* almost all ribosomal protein genes are lost from the mitogenome, but only some can be found in the nucleus.

## Introduction

Compared to the mitochondrial genome (mitogenome) of most other eukaryotes, the structure of the mitogenome of land plants is astonishingly dynamic. More than a hundred mitogenomes covering most major clades of land plants have now been completely sequenced, and particularly the more than 75 mitogenomes from angiosperms reveal an intriguing evolutionary history: changes in size, structure, gene and intron content, horizontal gene transfer (HGT) and transfer of DNA between genomic compartments, extensive RNA editing, extreme differences in substitution rates and frequent incorporation of retrotranscribed sequences (reviewed in [1, 2]).

Plant mitogenomes are usually shown as single, circular structures, but increasing evidence suggests that they can be multichromosomal with the individual structures taking diverse shapes, i.e. being circular, linear or branched [3]. Among angiosperms most mitogenomes have been depicted as 200–800 kb circular structures, however, the smallest being only 66 kb and the largest >11 Mb and assembled into more than 100 individual fragments [4, 5]. The ancestral angiosperm mitogenome is assumed to have included 41 protein coding genes: 20 encoding subunits of the respiratory chain complexes, 15 encoding proteins of the small and large ribosomal subunits, four involved in cytochrome c maturation, one encoding a translocase subunit and one encoding an intron maturase [1, 2]. Whereas the two *sdh* genes involved in the respiratory chain complex II and many of the ribosomal protein genes are recurrently lost, the remaining genes have been found in almost all angiosperms [1, 2, 6, 7]. Loss of protein coding genes from the mitogenome may occur following functional transfer of a gene to the nucleus [8]. In addition to protein coding genes, the mitogenome includes three ribosomal RNA genes universally present (possibly with the exception of *Viscum* [9]) and approximately 20 tRNA genes, most of which are frequently lost.

Import of foreign DNA is one of the mechanisms responsible for mitogenome size expansion. The most common source of foreign DNA is the plastome [2]. DNA of plastid origin has been found in all angiosperm mitogenomes investigated so far with various quantities ranging from 2–3 kb in *Arabidopsis*, *Silene* and *Vigna* to 113 kb in *Cucurbita* and 138 kb in *Amborella* [2, 10, 11]. Import of nuclear sequences to the mitogenome may occur but is difficult to detect. The availability of nuclear genomes is limited, and direction of potential transfers is not trivial to determine [2]. The most frequent nuclear sequences reported from angiosperm mitogenomes are transposable elements, though rarely in monocots, including the Alismatales [2, 12, 13]. Acquisition of foreign DNA through HGT is another means of size increase. HGT has been described primarily in parasitic plants, but the most extraordinary case is non-parasitic *Amborella*, where the mitogenome, through fusion with entire foreign mitogenomes, has experienced a roughly six-fold size increase [11, 14].

To address questions related to mitogenome evolution appropriate taxon sampling is needed (e.g., [4, 15–17]). In this study we focus on a clade of monocots, Alismatales, which several studies have shown to be a hotspot of mitochondrial anomalies, including pronounced intron loss, substitution rate variation, extremely different levels of RNA editing and potential abundant incorporation of retrotranscribed gene sequences [18–22]. The Alismatales includes three major clades: the Araceae, the Tofieldiaceae and a clade of 10 families here referred to as the core alismatids [22, 23]. The known mitochondrial anomalies are confined to the latter clade. By adding complete mitogenome sequences from two core alismatids, *Stratiotes aloides* (Hydrocharitaceae) and *Zostera marina* (Zosteraceae), to data already available [12, 13] we widen previous comparative studies of Alismatales mitogenomes [13]. Intergenomic transfer of sequences are explored using the complete nuclear genome sequence of *Zostera marina* [24] and the large amount of plastome gene data from Alismatales provided by Ross et al. [23]. Additionally, we provide gene sequence data from *Triantha* (Tofieldiaceae) and 27 species representing all families of the core alismatids. We will use these data to study the evolution of ribosomal protein genes in phylogenetic framework.

## Material and methods

### Mitochondrial genome sequencing, assembly and annotation

Specimens of *Zostera marina* L. (specimen voucher: A. Cuenca C2544, Denmark, Humlebæk Strand 6 Sept. 2009 (C)) and *Stratiotes aloides* L. (specimen voucher: O. Seberg et al. C2459, Denmark, Køge Bugt 10 Sept. 2008 (C)) were collected in the wild, and no specific permits were required for the collection of the material. The species are not protected by Danish law and they are collected in public areas where no permits are needed.

The complete mitogenomes were assembled using a combination of 454 and Illumina sequencing data. The 454 data generated from mitochondrial enriched DNA extractions, were re-used from Cuenca et al. [21]. For llumina sequencing, DNA was extracted from silica gel preserved material using a standard CTAB method [25]. Short-insert, paired-end (PE) libraries with average insert size of 500 bp were constructed and run in 1/16 of a lane on an Illumina HiSeq 2000 (Illumina, San Diego, CA). Libraries and sequencing data were produced at the Danish National High-Throughput DNA Sequencing Centre. Raw reads were trimmed for quality, adapters and unidentified nucleotides using AdapterRemoval [26].

454 reads were initially assembled in Newbler v. 2.3 (454 Life Sciences Corp, CT, USA) using default settings. Contigs were ordered using the program bb.454contignet (http://www.vcru.wisc.edu/simonlab/sdata/software/) that reads the assembling information from Newbler and depicts the contigs as a web, indicating contig connections [27]. Contigs were then extended and assembled as described by Cuenca et al. [13]. To identify reads of adjacent contigs and to determine the borders of duplications, contigs were extended by blasting approximately the last 75 nt of each contig border against a database of all raw 454 sequence reads. These BLAST analyses were done using the BLASTN program [28] in stand-alone BLAST ver. 2.2.21 (ftp://ftp.ncbi.nlm.nih.gov/blast/executables/blast+/LATEST/). Consensus sequences of each contig were used as seed sequences and extended using both 454 reads and Illumina reads in the Short Sequence Assembly by K-mer search and 3' read Extension program, SSAKE ver. 3.5 [29], with parameters -m 15 -o 2 -r 0.6 -p 0 -t 0 -v 1. Finally, all 454 and Illumina reads where mapped to the assembled mitogenomes using Geneious ver. 7.1 (Biomatters Ltd.) to verify the assemblies, evaluate coverage and correct for potential homopolymer length errors in particular attributable to the 454 reads.

Sequences of protein coding genes and rRNA genes were identified by BLASTN using a local database of extracted gene sequences from the 27 angiosperm species including *Butomus umbellatus* (NC021399 [13]) and *Spirodela polyrhiza* (NC0178840 [12]) from the Alismatales (S1 Table). In addition to complete protein coding genes with intact reading frames, fragments of known genes >100 bp were annotated. A sequence was recognized as a pseudogene if it had a length comparable to known functional genes, but could not be translated into an amino acid sequence even following potential RNA editing. The tRNA genes were identified using tRNAscan-SE 1.21 [30]. Annotation was performed manually in Geneious vers. 7.1–9.0 (Biomatters Ltd.). The assembled and annotated genomes are deposited in GenBank under accession numbers KX808393 (*Stratiotes aloides*) and KX808392 (*Zostera marina*).

To verify deviating gene sequences in the mitogenome of *Zostera marina*, we produced Illumina sequences for another species of *Zostera*, *Z*. *noltii* Hornem. (specimen voucher: Seberg et al. C2453, Denmark, N of Munkholmbroen 10 Sept. 2008 (C)). DNA extraction and sequencing were performed as above, except that the library was run on an Illumina HiSeq 2500. Complete assembly of this mitogenome was not attempted, but the genes except the tRNA genes, were extracted after mapping the Illumina reads to the *Zostera marina* mitogenome using Geneious ver. 8.0 (Biomatters Ltd.). Reads mapping to identified, complete or partial genes were used for *de novo* assembly of individual loci, and if necessary in order to obtain complete gene sequences one or more rounds of Map to Reference as implemented in Geneious ver. 8.0 (Biomatters Ltd.) were used to extend the sequence of individual loci. A local database of genes from other completely assembled mitogenomes (S1 Table) was used as reference sequences to search for genes not present in *Z*. *marina*. Identified genes and flanking sequence of *Z*. *noltii* are deposited in GenBank under accession numbers KX808258-KX808296.

### Sequence analysis

To identify regions of potential plastid origin in the complete mitogenomes of *Zostera* and *Stratiotes* we performed a BLASTN search against a database of 23 angiosperm plastid genomes, including genomes from the genera *Elodea* (Hydrocharitaceae), *Lemna* and *Spirodela* (Araceae) from the Alismatales (S1 Table). Plastid genome sequences of *Stratiotes* and *Zostera* are not available. Only mitochondrial sequences larger than 100 bp and with a similarity score higher than 80% were considered. If the hits included protein coding gene sequence we used the mitochondrial sequence matching the plastid protein coding gene to perform a new BLASTN search against all sequences in GenBank and against local databases of plastid gene sequences created from the data provided by Ross et al. [23] available at figshare.com (DOI: 10.6084/m9.figshare.1407422.v1). We also included the protein coding sequences in phylogenetic analyses using the matrices from Ross et al. [23]. We realigned the sequences after inclusion of the new sequence copies found in the mitogenomes using the MUSCLE [31] plugin in Geneious ver. 8.1. Subsequent phylogenetic analyses were performed using RAxML ver. 7.2.8 [32] with 100 replicates of rapid bootstrapping and a GTR+GAMMA+I model as implemented in Geneious ver. 8.1.

To identify regions of potential nuclear origin in the mitogenomes of *Zostera* and *Stratiotes* we searched for repetitive elements using the Repbase Update repetitive element database [33]. For *Zostera* we also performed a BLASTN search (maximum E-value = 1e-50) of the complete mitogenome against a local database created in Geneious ver. 9.0 including all the contigs from the *Zostera marina* nuclear genome (GenBank no. LFYR00000000 [24]). BLASTN results of sequences longer than 250 bp and a pairwise similarity >80% were inspected for sequence features, which could indicate the direction of potential transfers. E.g., finding a sequence in the mitogenome including (part of) a gene normally located in the nuclear genome would indicate transfer from the nuclear genome to the mitogenome.

We searched for dispersed repeated sequences in the complete mitogenomes of *Zostera* and *Stratiotes* by blasting the sequences against themselves using BLASTN. The results were filtered to retain only matches of sequences longer than 100 bp and pairwise similarity >80%.

To evaluate the conservation of gene order in the Alismatales, we searched for clusters of genes shared between the complete mitogenomes of *Spirodela*, *Butomus*, *Zostera* and *Stratiotes*. Gene clusters are defined as two or more adjacent genes in the same direction shared by at least two taxa. This was done by simple visual inspection of the annotated mitogenome sequences.

To get a rough estimate of mitogenome DNA similarity between pairs of Alismatales species we made pairwise BLASTN analyses with the four complete mitogenome sequences. Due to the heuristic nature of BLAST, blasting a sequence A against B, does not necessarily give the same result as blasting B against A. Accordingly, all analyses were done in both directions and average values were calculated as in Guo et al. [34].

### Ribosomal protein genes in Alismatales

Illumina sequencing data from *Triantha* and 26 core alismatids provided by Ross et al. [23] were used to retrieve ribosomal protein coding genes as we previously retrieved other protein coding genes from the same data [21]. The sequences are deposited under accession numbers KX808234-KX808257 and KX808297-KX808391. Assuming that genes can be lost, but not gained (see below), we mapped loss of ribosomal protein genes on a phylogenetic tree of the Alismatales using Dollo parsimony. We used a tree modified from the result of a total evidence analysis based on morphology, plastid and mitochondrial data (Figure 1 in [22]). The tree was modified by reducing the taxon content to match the present sampling and by assuming that the relative positions of *Spirodela* and *Triantha* are the same as for the representatives of Araceae and Tofieldiaceae, respectively, as used by Petersen et al. [22].

The nuclear genome of *Zostera* [24] includes several NUMTs (nuclear mitochondrial sequences) that contain mitochondrial ribosomal protein genes. We compared the ribosomal protein NUMTs by BLASTN to a local database including the Alismatales mitochondrial gene sequences plus sequences from up to 50 other angiosperms (S1 Table), and to assess the origin of two NUMTs we performed phylogenetic analyses using the same sequences as included in the local databases. The sequences were aligned using the default settings in MUSCLE [35] as implemented in Geneious ver. 9.0. Subsequent phylogenetic analyses were performed using RAxML ver. 7.2.8 [36] with 100 replicates of rapid bootstrapping and a GTR+GAMMA+I model as implemented in Geneious ver. 9.0.

## Results

### Mitogenome size and gene content of *Stratiotes* and *Zostera*

**Fig 1:**
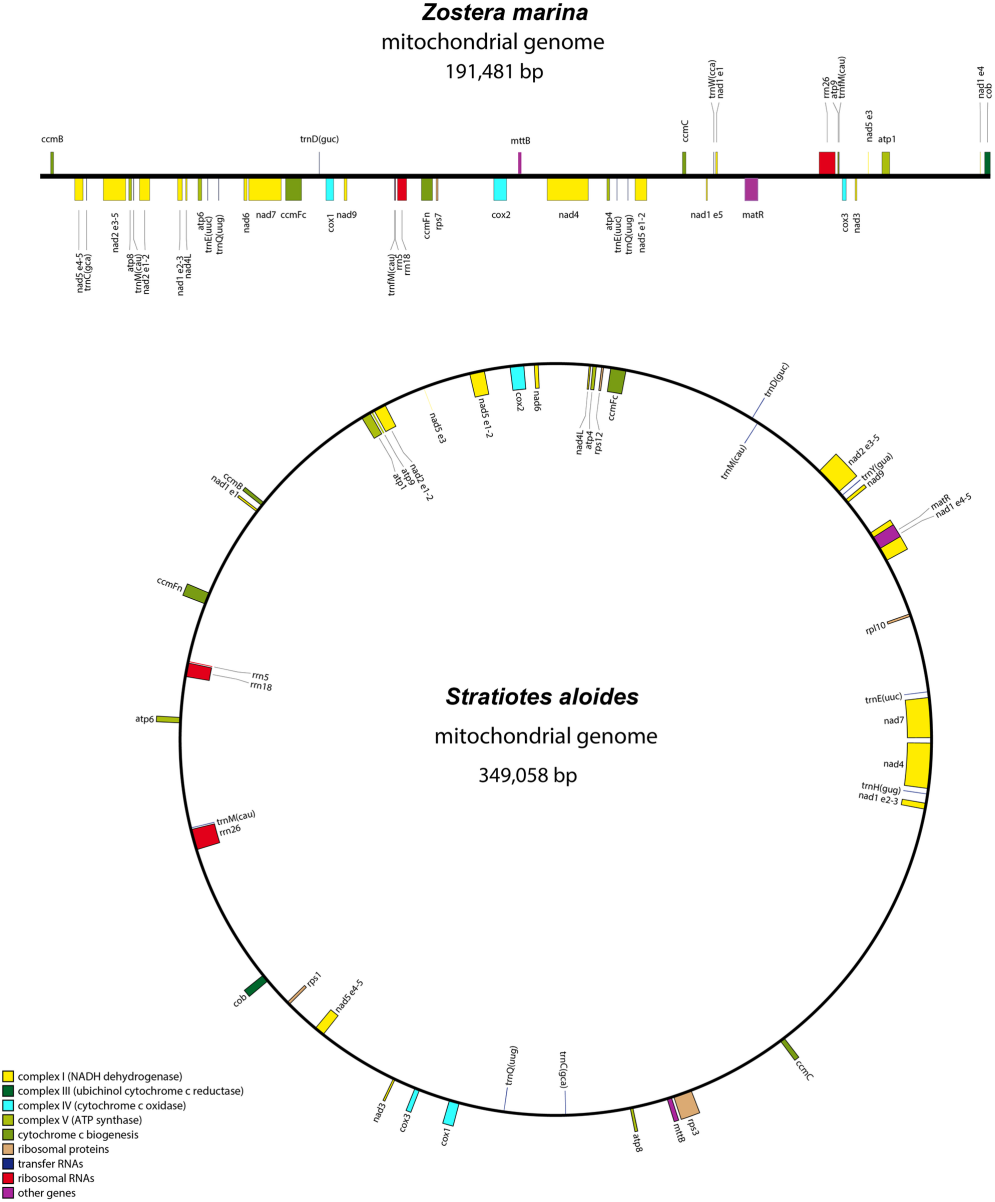
Maps of mitogenomes of *Zostera marina* and *Stratiotes aloides*. For *Zostera* upper genes are transcribed forward; lower genes are transcribed reverse. For *Stratiotes* inner genes are transcribed clockwise; outer genes are transcribed counter-clockwise. Genes are color coded as indicated in the lower left corner. The names of *trans*-spliced genes are followed by exon numbers. Only genes assumed to be functional are shown. The figure was created using OGDRAW [38].

The *Stratiotes aloides* mitogenome could be assembled into a 349,058 bp circular structure whereas the mitogenome of *Zostera marina* was assembled into a shorter linear structure of just 191,481 bp (Fig 1, Table 1). Details of read numbers and coverage are listed in S2 Table. The large number of repeats in *Zostera* makes numerous submolecular structures possible, but we failed to map reads into a master circle unless accepting a duplication of more than 68 kb, which is not supported by read coverage. Although larger duplications have been reported [37], we prefer to draft a linear genome.

**Table 1:** Characteristics of mitogenomes of four representatives of the Alismatales.

	MT size (bp)	CP inserts	GC% Total/MT/CP^a^	Repeats	Gene DNA^b^	Protein genes^c^	Introns^d^	tRNAs^c^	rRNAs^c^
*Spirodela*^e^	228,493	4.1%	45.7/-/-	3.2%	57,500	35	23(6)	15	3
*Butomus*^f^	450,826	1.5%	49.1/49.1/50.2	8.3%	63,044	29^g^	22(5)^h^	9	3
*Stratiotes*	349,058	14.2%	45.2/46.2/39.3	0.6%	58,789	28	22(5)	8	3
*Zostera*	191,481	20.5%	45.1/46.6/39.3	≈ 20%	55,703	25	21(5)	6	3

^a)^ GC content in the complete mitogenome and split into the fractions of mitochondrial and plastid origin

^b)^ Length of DNA (bp) in all genes and *cis*-spliced introns

^c)^ Genes in CP inserts not included

^d)^ Total number (*trans*-spliced)

^e)^ Data from Wang et al. [12]

^f)^ Data from Cuenca et al. [13]

^g)^ *rpl10* has been detected after the publication of Cuenca et al. [13]

^h)^ *cox2* exon 3 has been detected after the publication of Cuenca et al. [13]

Mitogenome characteristics are summarized in Table 1, and a complete list of genes including length information is provided in S3 Table. With regard to gene content, the two new complete genomes differ in their content of ribosomal protein genes and tRNA genes, only. *Stratiotes* has four ribosomal protein genes (*rpl10*, *rps1*, *rps3*, *rps12*) and two pseudogene sequences (*rpl16*, *rps7*), whereas *Zostera marina* only has one complete gene (*rps7*) and a fragments of another (*rps3* exon2). In *Zostera noltii* two *rps3* fragments were detected (S3 Table). *Stratiotes* also has more tRNA genes than *Zostera marina*. A total of 11 tRNA genes normally found in plant mitogenomes were detected in *Stratiotes* (S3 Table), however three of them are located in regions assumed to be recently transferred from the plastome (see below). In *Zostera marina* we found 8 tRNA genes, two of which are of recent plastid origin (see below).

The protein coding genes of *Stratiotes* and the two species of *Zostera* differ slightly with regard to introns (Tables 1 and 2). Due to the presence of *rps3* (which includes a *cis*-spliced intron) in *Stratiotes* but not in *Zostera*, the total number of introns differs (21 vs. 22), and in *Stratiotes* nad1i728 is *cis*-spliced but *trans*-spliced in *Zostera*. For two of the protein coding genes (*nad1* and *nad4L*) the start codon is created by RNA editing in both *Stratiotes* and *Zostera*. In *Zostera* editing is confirmed by comparison to the EST library data from *Zostera marina* (GenBank, SRR057351). In *Zostera* an alternative start codon of *atp9* and *cob* may be used, possibly GTG in *atp9* and ATA in *cob*.

**Table 2:** Intron content (*cis-* and *trans*-spliced) in mitogenomes of the Alismatales.

	*Spirodela*	*Butomus*	*Stratiotes*	*Zostera*
ccmFci829	●	●	●	●
cox2i373^a^	●	−	−	−
cox2i691^b^	●	●	●	●
nad1i394	**ө**	**ө**	**ө**	**ө**
nad1i477	●	●	●	●
nad1i669	**ө**	**ө**	**ө**	**ө**
nad1i728	**ө**	●	●	**ө**
nad2i156	●	●	●	●
nad2i542^c^	**ө**	**ө**	**ө**	**ө?**
nad2i709	●	●	●	●
nad2i1282	●	●	●	●
nad4i461	●	●	●	●
nad4i976	●	●	●	●
nad4i1399	●	●	●	●
nad5i230	●	●	●	●
nad5i1455	**ө**	**ө**	**ө**	**ө**
nad5i1477	**ө**	**ө**	**ө**	**ө**
nad5i1872	●	●	●	●
nad7i140	●	●	●	●
nad7i209	●	●	●	●
nad7i676	●	●	●	●
nad7i917	●	●	●	●
rps3i74^d^	●	●	●	n.a.
total *cis* (●)	17	17	17	15
total *trans* (ө)	6	5	5	6

^a)^ This intron is only present in *Spirodela*

^b)^ This intron was not recognized previously in *Spirodela* and *Butomus* where the short exon 3 was overlooked [12, 13]

^c)^ Splicing of this intron in *Zostera* is discussed in the text

^d)^ N.a. = non applicable. *Zostera* lacks *rps3*

### Dispersed repeats in *Stratiotes* and *Zostera*

The two new, complete mitogenomes differ considerably both in number and pattern of repeated sequences (Table 1, S4 Table). *Stratiotes* has only 12 repeated sequences longer than 100 bp, and the longest is just 382 bp, thus accounting for only approximately 0.6% of the entire mitogenome. In *Zostera* 209 repeats were detected, the longest more than 10 kb. However, a number of the suggested repeats in *Zostera* are overlapping, representing different estimates of the repeats with just slightly different lenghts. Thus, the true number of repeats is lower than 209. Due to this ambiguity, the proportion of repeated sequences in the genome is also difficult to assess precisely, but we estimate that repeats account for approximately 20% of the genome.

### Insert of foreign DNA in the mitogenomes of *Stratiotes* and *Zostera*

The mitogenomes of both *Stratiotes* and *Zostera* include a substantial amount of plastid DNA. In *Stratiotes* 11 fragments ranging in size from 641–12,253 bp with a total length of 49,392 bp (corresponding to 14.2% of the mitogenome) were detected, and in *Zostera* 27 fragments in the range of 140–6,614 bp, totaling 39,563 bp (corresponding to 20.5% of the mitogenome), were found (Table 1, S5 Table). Disregarding sequences that are repeated in the mitogenomes, the inserts in *Zostera* and *Stratiotes* correspond to 31,678 bp and 50,261 bp of the complete plastome of *Elodea canadensis* (NC018541), respectively. For the previously published mitogenome of *Butomus*, we calculated that the 6,928 bp of plastid inserts correspond to 7,235 bp of the *Elodea* plastome (S5 Table).

Results from blast searches and phylogenetic analyses of inserted plastid sequences are listed in S6 Table. The gene trees are not shown, but the placement and bootstrap support values for the relevant clade including the transferred plastid sequence are listed. For both *Stratiotes* and *Zostera marina* almost all inserted gene sequences, and in particular those included in long inserts, are most similar to sequences from *Stratiotes* and *Zostera muelleri* (the data set from Ross et al. [23] used for similarity searches and phylogenetic analyses includes *Z*. *muelleri* instead of *Z*. *marina*), respectively, and the sequences are grouped together in the gene trees (not shown). Noteworthy exceptions are two inserts in *Stratiotes*: a 931 bp insert including a partial sequence of *ndhB* that is most similar to *Potamogeton* (Potamogetonaceae) and placed in the gene tree as sister to a clade including Ruppiaceae, Cymodoceaceae, Zosteraceae and Potamogetonaceae, and a 569 bp insert including a partial *ndhK* sequence that is most similar (70.3%) to *Illicium* (Schisandraceae) and placed together with a transferred gene sequence from *Butomus* within early diverging angiosperm lineages in a gene tree. In contrast to the plastid inserts in *Zostera* and *Stratiotes*, only the longest insert (4,897 bp) in *Butomus* seems to stem from intraspecific, intergenomic transfer. The remaining inserts, including mostly very short gene fragments, show similarity and weakly supported or unsupported relationships to different groups of angiosperms (S6 Table).

Insertion of nuclear DNA could be most precisely investigated in *Zostera marina* where the complete nuclear genome has been sequenced [24]. Blasting the complete mitogenome sequence of *Zostera* against the nuclear genome resulted in 329 matches fulfilling the search criteria. A total of 57 matches included sequences >250 bp long and with a similarity >80% (S7 Table). Of these, 17 matches involve plastid sequences, 25 involve sequences with part of or rarely a complete mitochondrial gene, and the remaining do not include sequences with any particular features. Eight matches between highly similar sequences involve an approximately 38 kb long sequence of the mitogenome and approximately 22 kb of the nuclear scaffold 54 (LFYR01001529). The 38 kb mitogenome sequence includes several complete genes (*trnM*, *rrn5*, *rrn18*, *ccmFn*, *rps7*, *cox2*, *mttB*), a large fraction of *nad4* and five regions of plastid sequence, but the nuclear scaffold has some long assembly gaps, e.g., where plastid regions, *rrn18* and *cox2*, should be located according to the mitogenome sequence.

A search for nuclear repetitive elements in both the *Zostera* and *Stratiotes* mitogenome retrieved no convincing positive results. Though the search criteria used here suggested similarity (82–96%) of three short sequences (121–310 bp) from each of *Zostera* and *Stratiotes* to repetitive elements, the sequences are all more similar to part of a chloroplast insert (92–100%) or to a mitochondrial rRNA gene (100%), and thus do not likely originate from repetitive elements.

### Mitogenome similarity and gene clusters in Alismatales

Mitochondrial DNA similarity between pairs of species of Alismatales is given in Table 3. *Butomus* and *Stratiotes* share the greatest amount of highly similar DNA (112 kb) whereas the least is found in a comparison between *Spirodela* and *Zostera* (58 kb).

**Table 3:** Mitochondrial DNA similarity between pairs of species of Alismatales.

	*Spirodela*	*Butomus*	*Stratiotes*	*Zostera*
*Spirodela*	−	90.2/82.3	80.2/73.1	61.3/54.8
*Butomus*	86	−	111.7/112.7	67.0/63.1
*Stratiotes*	77	112	−	74.7/69.8
*Zostera*	58	65	72	−

Numbers (kb) above the diagonal are from reciprocal BLASTN analyses, and numbers below the diagonal are average values.

Gene order is conserved very poorly in Alismatales. Only 11 colinear gene clusters including two or three genes were found (Table 4). A single cluster (*rrn18-rrn5*) is shared by all four taxa, six are shared by three taxa, but only one of them (*nad3-cox3*) by the core alismatids.

**Table 4:** Colinear gene clusters in four representatives of the Alismatales.

	*Spirodela*	*Butomus*	*Stratiotes*	*Zostera*
*nad4L-atp4*	+	+	+	-
*rps3-rpl16*	+	+	+	-
*trnfM-rrn26*	+	+	+	-
*rrn18-rrn5*	+	+	+	+
*trnY-nad2*.e3+4+5	+	+	+	-
*nad9-trnY*	+	+	+	-
*nad1*.e4-*matR-nad1*.e5	--	++	++	--
*cox2-nad6*	-	+	+	-
*atp1-atp9-nad2*.e1+2	--	++	++	--
*nad3-cox3*	-	+	+	+
*nad4-trnH*	-	+	+	-

Clusters above the dotted line are possibly ancestral to the angiosperms [15]. Plus/minus symbols indicate presence/absence of a gene cluster. Two symbols are used to indicate presence/absence of individual gene pairs in clusters of three genes.

### Loss and transfer of ribosomal protein genes in *Zostera* and other core alismatids

**Fig 2:**
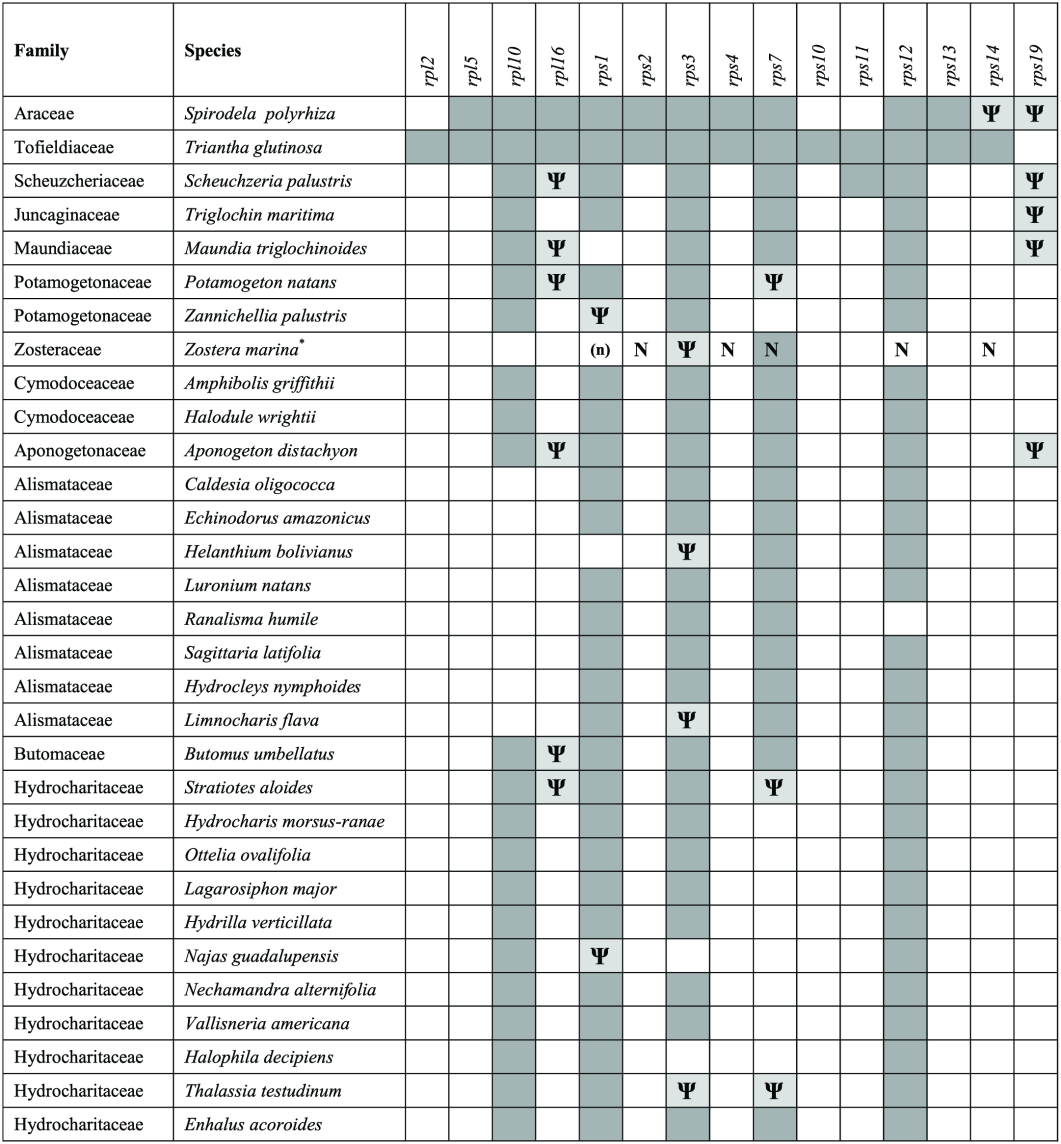
Distribution of mitochondrial ribosomal protein genes in Alismatales mitogenomes. Blank boxes indicate absence of a gene from the mitogenome. Dark grey boxes indicate presence of a gene assumed to be functional. Light grey boxes and **Ψ** indicates a pseudogene or fragment of gene in the mitogenome. **N** indicates presence of a gene annotated as of mitochondrial origin in the nuclear genome of *Zostera marina*. (**n**) indicates likely presence of a gene in the nuclear genome of *Zostera marina*, though not annotated as of mitochondrial origin. *Zostera noltii has the same mitochondrial genes, but the potential nuclear location of genes is unknown.

**Fig 3:**
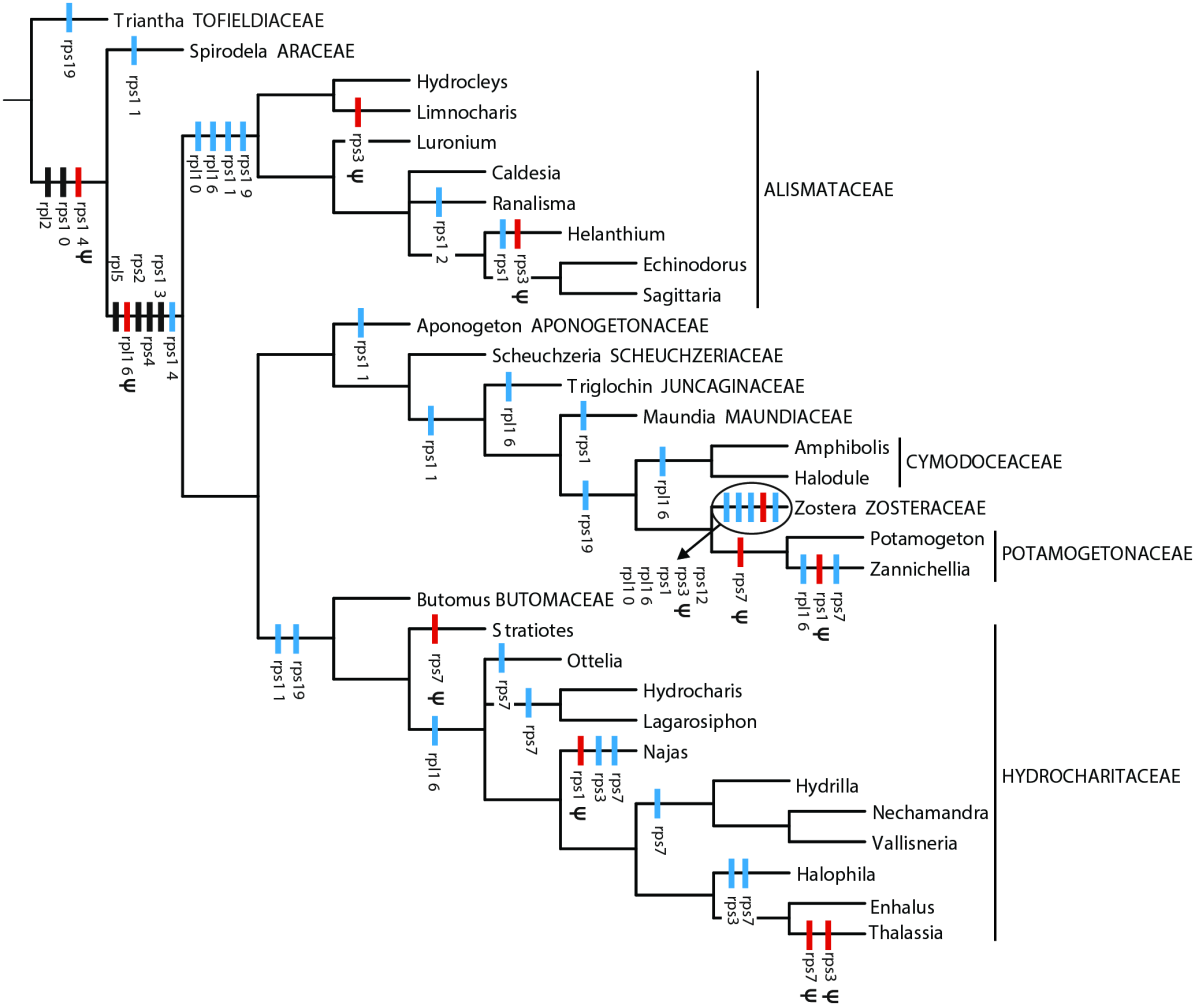
Loss and pseudogenization of mitochondrial ribosomal protein genes in Alismatales. Bars represent events of loss or pseudogenization. Black bars mark unique events of gene loss from the mitogenome. Red (pseudogenization) and blue (loss) bars mark homoplasious gene changes.

A survey of ribosomal protein genes in 31 representatives of Alismatales revealed a substantial loss of genes from the mitogenome in all core alismatids (Fig 2). *Triantha* has an almost full complement with 14 ribosomal genes, but only one (in *Zostera*) to six genes (in *Scheuzcheria*) were found in the core alismatids. An evolutionary hypothesis about gene loss and pseudogenization is created by mapping the events on a phylogeny of the Alismatales assuming that genes may be lost but not gained, and that pseudogenes do not revert to functional copies (Fig 3). Nine genes (*rpl2*, *rpl5*, *rpl16*, *rps2*, *rps4*, *rps10*, *rps13*, *rps14*, *rps19*) are lost only once either completely or first functionally by pseudogene formation. The remaining six genes have been lost at least twice.

In the nuclear genome of *Zostera marina* five genes (*rps2*, *rps4*, *rps7*, *rps12* and *rps14*; NCBI; LFYR00000000) are annotated as ribosomal protein genes of mitochondrial origin (NUMTs). Three of them (*rps2*, *rps4*, *rps14*) are missing from the mitogenome of all core alismatids, and the similarity of the NUMTs in *Zostera* to the mitochondrial genes in *Triantha* and *Spirodela* is small (approximately 40–60%). The *rps12* gene is missing from the mitogenome only in *Zostera* and *Ranalisma* (Alismataceae). In most Alismatales the coding sequence of *rps12* includes one reading frame of 378 bp, whereas the nuclear *Zostera* gene is split in two exons of 133 bp and 377 bp (scaffold 89, LFYR01002109), the second corresponding to the mitochondrial gene. Similarity between the nuclear *rps12* exon2 and mitochondrial *rps12* sequences is close to 75% for all Alismatales, and the phylogenetic analysis place the NUMT with very weak support (30% bootstrap) as the sister to a clade including *Maundia*, *Triglochin*, *Halodule*, *Amphibolis*, *Potamogeton* and *Zannichellia*. The mitochondrial *rps7* gene is missing or pseudogenized in some core alismatids, but present in *Zostera*. The *rps7* NUMT, which is located in scaffold 54 (LFYR01001529), is included in the 22 kb region described above and the gene sequence is identical to the sequence of the mitochondrial gene copy. Transcriptome data from *Z*. *marina* available in GenBank (SRX021650) indicate that *rps7* transcripts are C to U edited, hence stemming from a mitochondrial gene.

A BLASTN search for the remaining mitochondrial genes both missing from the *Zostera* mitogenome and not annotated in the nuclear genome was performed with genes from other alismatids as templates. This gave a match to a full length coding sequence (480 bp) 81% similar to the *rps1* gene in *Potamogeton*. The potential NUMT is included in a larger nuclear region annotated as a hypothetical protein (LFYR01001306, ZOSMA_440G00060). A phylogenetic analysis of the NUMT places it with very weak support (44% bootstrap) as sister to *Triglochin* (Juncaginaceae), however, the group is strongly supported within a clade also including the Cymodoceaceae (98% bootstrap). Another BLAST match (85.2% similarity) was found between the mitochondrial *rpl10* gene (279 bp) from *Potamogeton* and a short nuclear sequence (169 bp) in *Zostera*. The nuclear sequence is part of a larger annotated hypothetical protein gene (LFYR01001739, ZOSMA_65G01050), but beyond the region with BLAST match the sequence has very limited similarity to *rpl10* genes, even at the amino acid level.

## Discussion

### Mitogenome size evolution

In general, mitogenomes of Alismatales are relatively small in overall size (bp) compared to other angiosperms (see e.g., [2]). The genome of *Stratiotes* (349,058 bp) lies in between the previously sequenced *Butomus* (450,826 bp) and *Spirodela* (228,493 bp) genomes whereas the mitogenome of *Zostera* (191,481 bp) is the smallest of all autotrophic angiosperms sequenced so far. Until now a smaller plant mitogenome (65,873 bp) has only been described in the hemiparasite *Viscum scurruloideum* [5], but despite *Viscum* mitogenomes being considerably and consistently reduced in gene content, they are not consistently reduced in size. The mitogenome of *V*. *album* has a rather standard size of 565,432 bp [9]. With only four completely sequenced mitogenomes from Alismatales it is impossible to provide a comprehensive picture of size evolution, however, both expansions and reductions have occurred. Considering the major differences observed in mitogenome size even between other closely related taxa, e.g., in the genus *Silene* and the family Cucurbitaceae [4, 10, 39], a general trend is not to be expected, rather, mitogenome size tends to be idiosyncratic across the land plants. Christensen [40, 41] proposed that different mechanisms of DNA repair in coding and non-coding regions of plant mitogenomes could explain the co-occurrence of low mutation rate in the coding regions and high variability, including size expansion, of non-coding regions. However, the mechanism based on a model of inaccurate double-strand breaks proposed for non-coding regions seems insufficient to explain size reductions, which most likely have occurred also in other angiosperm lineages, e.g., the Brassicales [16].

Consistent with a lack of a general pattern of size evolution, DNA similarity between Alismatales mitogenomes is limited (Table 3). Though the two most closely related genera, *Butomus* and *Stratiotes*, do share the greatest amount of highly similar DNA, this corresponds to only a fourth or a third of the complete mitogenomes. The approximately 112 kb of shared, similar DNA is about twice as much as the amount of genic sequence (incl. *cis*-spliced introns) in the two genomes (approximately 59 kb and 63 kb), thus conserved regions also involve non-coding sequences. In contrast, the lowest amount of shared, similar DNA between any pair of species, viz. *Zostera* and *Spirodela* (approximately 58 kb), corresponds very closely to the amount of gene sequence in *Zostera* (approximately 56 kb), showing that virtually all non-gene DNA has either been replaced or shuffled beyond conventional limits of similarity recognition.

Mitogenome size can be positively affected by the occurrence of duplicated sequences as most dramatically seen in *Silene conica* where more than 40% (approximately 4,600 kb) of the mitogenome is composed of repetitive DNA [4]. The three core alismatid mitogenomes do differ considerably in amount and pattern of repeated sequences (Table 1, S4 Table), and in the two closest relatives, *Butomus* and *Stratiotes*, repeated sequences account for approximately 8.3% and 0.6% of the mitogenomes, respectively. Thus, slightly more than 1/3 of the approximately 100 kb size difference between *Butomus* and *Stratiotes*, (i.e., 37.8 kb according to Cuenca et al. [13]), can be explained by sequence duplications in *Butomus*. Surprisingly, the smallest Alismatales mitogenome, the approximately 191 kb *Zostera* genome, has both the highest number of repeated sequences and the largest total amount of repeated DNA, accounting for approximately 20% of the total size. Thus, as previously observed, e.g., in the asterid lineage and in *Silene* [4, 42], there appears to be no clear correlation between mitogenome size and content of repeated sequences.

### Foreign DNA

Mitogenome size may also be affected by the incorporation of foreign DNA either by intracellular transfer from the plastid or nuclear genome or from horizontal transfer of mitochondrial DNA. The mitogenomes of the four Alismatales all include plastid DNA within the range observed in other angiosperms [2], however, with the smallest (approximately 7 kb) and largest (approximately 49 kb) amounts found in the closest relatives, *Butomus* and *Stratiotes* (S5 Table). The relative amount of plastid DNA in the mitogenome of *Butomus* (1.5% [13]) is among the lowest yet observed in any angiosperm, where smaller fractions (0.8% and 1.1%) have only been reported in species of *Gossypium* [17]. Thus, more surprising is the discovery of almost 40 kb of plastid DNA in the mitogenome of *Zostera*, accounting for more than 20% of its total size. This relative fraction by far exceeds the previously reported highest relative amounts of approximately 10–12% in *Boea* and *Cucurbita* [10, 43].

The data for plastid inserts suggest a correlation between quantify of inserted DNA, length of inserts and the relative timing of insert events. The large amount of plastid DNA in *Zostera* and *Stratiotes* is coupled with the presence of several long sequences, which are transferred recently. Recent transfer is suggested both on the basis of raw sequence similarity to congeneric plastid sequences and by phylogenetic analyses (S6 Table). However, without a denser taxonomic sampling of comparable plastome sequences any absolute timing of transfer events cannot be estimated meaningfully, so we just conclude that these transfers have taken place after divergence of each of the two genera from their respective sister groups. In contrast, *Butomus* includes only a single long insert of such recent origin. A single insert sequence (including the *ndhK* gene) is found in both *Stratiotes* and *Butomus* and our data suggest that it may have an origin among early angiosperms (S6 Table). Inserts including the *ndhK* gene have been found in several other angiosperms including *Amborella*, *Liriodendron* and many eudicots and monocots e.g., [11, 15, 43, 44]. This, however, does not support a scenario of one ancient transfer event, as almost all the inserted sequences are more similar to their conspecific or congeneric plastome gene copies than to each other (data not shown). Thus, repeated transfer events seem more likely, unless conversion between plastome and mitogenome sequences as suggested by Clifton et al. [44] also has occurred repeatedly. In both *Amborella* and *Liriodendron* the inserts including *ndhK* are longer (>5 kb and >1 kb, respectively) [11, 15] than those we find in *Stratiotes* and *Butomus*, thus it remains a possibility that the latter sequences are remnants of a longer, ancient transfer.

Although both *Zostera* and *Stratiotes* include large fractions of plastid DNA of recent origin there are some differences. In *Zostera*, the total amount of plastid DNA is less than in *Stratiotes* and the sizes of the inserts are generally smaller, too. However, the number of inserts is larger, suggesting that transfer events either occur more frequently or that the original inserts have been fragmented subsequently (S5 Table). The high number of short non-overlapping plastid inserts, including three non-overlapping inserts of the *rpoB* gene, suggests that fragmentation and rearrangements have shaped the *Zostera* mitogenome particularly rapidly compared to both *Stratiotes* and *Butomus*.

Based on the data from *Butomus*, Cuenca et al. [13] previously suggested that the transfer of protein coding sequences from the plastid to the mitochondrial genome possibly took place through reverse transcription. The current data from *Zostera* and *Stratiotes*, which include long inserts with several genes that are not likely to be co-transcribed, do not support that hypothesis. A scenario of frequent intracellular transfer events of longer pieces of DNA followed by rapid fragmentation and rearrangement as previously inferred in other plants [44–46] seems more likely.

Intracellular transfer of DNA between the nuclear and mitochondrial genomes may also shape both genomes. However, due to lack of features in larger parts of both the nuclear genomes and mitogenomes, the direction of a potential transfer can be difficult to assign even in the few cases where the nuclear genome has been sequenced [2]. With the complete nuclear genome of *Zostera marina* being available [24] a full comparison of the mitogenome and the nuclear genome was possible, and some intracellular transfer has clearly taken place. Some similar or even identical sequences include mitochondrial genes, thus most likely they are the result of transfer from the mitogenome to the nuclear genome. Most prominent among these is a transfer of an approximately 38 kb region of mitochondrial DNA including several complete genes (S7 Table). Based on the highly conserved sequence, the transfer most likely occurred very recently—possibly after divergence of *Z*. *marina* from its sister species. The mitochondrial sequence contains inserts of plastid origin, but the NUMT has annotation gaps at the exact location of the plastid inserts. The most simple explanation is that the plastid sequences were transferred to and incorporated into the mitogenome first, and subsequently the entire region was transferred to the nucleus. Either due to assembly ambiguity caused by sequence similarity between genomic fractions or because plastid reads may have been filtered out, the transferred plastid sequences were not correctly incorporated in the nuclear genome sequence.

### GC content

The mitogenomes of *Stratiotes* and *Zostera* are very similar with regard to GC content, 45.2% and 45.1%, respectively (Table 1). While these values are completely normal compared to *Spirodela* (45.7%) and other angiosperms, which usually have a GC content in the range of 43–45% [2, 13], they are in contrast to the increased GC level previously found in *Butomus* (49.1%) [13]. The GC content of a mitogenome may be affected by recent transfer of DNA from the plastome, which is generally characterized by a low GC content [43, 46]. Both *Stratiotes* and *Zostera* have rather large amounts of plastid DNA in their mitogenomes (see above), which bias the total GC content as the plastid inserts in both mitogenomes have a GC content of only 39.3% (Table 1), very close to the 39.0% GC content estimated by Sloan and Wu [46] for ancestral plastid sequences transferred to a diverse range of angiosperm mitogenomes. The GC contents of the remaining part of these two core alismatid mitogenomes are 46.2% and 46.6%, respectively, thus in the high end of the usual spectrum, but still not as high as in *Butomus*, where the total GC content is unaffected by the small amount of plastid DNA. The GC content of the few plastid inserts in *Butomus* is even slightly higher (50.2%) than that of the remaining mitogenome, consistent with the inserts primarily being older and thus having been brought into balance with the rest of the mitogenome [46, 47].

### Gene content and gene features

The gene contents of *Stratiotes* and *Zostera* are normal compared to other Alismatales and to angiosperms in general with regard to ribosomal RNA genes and protein coding genes, with the exception of the ribosomal protein genes (Table 1, S3 Table). Excluding the variation in ribosomal protein gene content, the only protein coding gene that is not consistently present is *sdh4*, which is absent from the mitogenomes of the three core alismatids. Together with *sdh3*, which is also lost in all Alismatales, the *sdh4* gene is frequently missing in angiosperms [7, 48]. The number of tRNA genes in the core alismatids is relatively low compared to *Spirodela* and most other angiosperms (see e.g., [4, 13]), however, plant mitochondria are capable of importing cytosolic tRNAs encoded in the nucleus [49, 50] and all genes appear to be dispensable.

The Alismatales is characterized by extreme variation in the occurrence of introns in the protein coding genes, with only six cis-spliced introns retained in some members of the Hydrocharitaceae [21]. However, the species investigated in the present study are among those with a quite normal array of introns compared to other angiosperms [51], and only minor differences exist among them (Table 2). A few differences in intron splicing are also observed. In *Butomus* and *Stratiotes* nad1i728 is *cis*-spliced, but it is *trans*-spliced in *Spirodela* and *Zostera*. In light of the phylogeny, the simplest evolutionary explanation is a change from *trans* to *cis*-splicing, however, all evidence from other plant mitochondria suggests that evolution proceeds only from *cis-* to *trans*-splicing [1, 52]. The nad1i728 intron has changed from *cis-* to *trans*-splicing several times during angiosperm evolution [1, 53], and could have done so in the Alismatales, too. However, a second intron, nad2i542 in *Zostera marina*, may suggest that a change from *trans-* to *cis*-splicing is possible. This intron is *cis*-spliced in *Isoetes* and *Selaginella*, but *trans*-spliced in all seed plants [49]. In *Zostera marina* we find the two flanking exons just 2,764 bp apart, which is a normal length for a group II intron. The region includes both another protein coding gene, *atp8*, and a tRNA gene, and the entire configuration may be the result of random events of rearrangement. Thus, it is entirely possible that nad2i542 is still *trans*-spliced, but the proximity of the *nad2* exons poses the obvious question, whether it is possible to regain a functional *cis*-spliced intron.

Richardson et al. [15] describe the distribution of 29 colinear gene clusters among angiosperms, 17 of which were assumed to be ancestral in the angiosperms and an additional three are supposed to be ancestral in the monocotyledons. Richardson et al. [15] did not include the intron splicing maturase gene, *matR*, which is located within nad1i728 in many angiosperms as well as other vascular plants, mosses and hornworts [54]. Fifteen of these clusters are present in *Spirodela*, but a number of the clusters include genes which are not present in the core alismatids (ribosomal genes and tRNA genes mainly), thus only six of the gene clusters are present in *Butomus* and *Stratiotes* and only the universally occurring *rrn18-rrn5* gene cluster is also present in *Zostera*. Among the Alismatales five new gene clusters not present in *Spirodela* are shared between *Butomus* and *Stratiotes*, only one of them also shared with *Zostera*. Though gene order is highly variable even among closely related species or within a species [17, 37], the quantity of rearrangements having shaped the *Zostera* mitogenome is unique.

### Mitochondrial ribosomal protein genes in the Alismatales

A survey of mitochondrial ribosomal protein genes in 280 angiosperms identified the Alismatales as a hot spot for gene loss [7], and the present data confirms this (Fig 2). Whereas *Triantha* (Tofieldiaceae), representing the sister group to *Spirodela* (Araceae) and the core alismatids, has an almost full complement of ribosomal protein genes, lacking only *rps19*, and *Spirodela* has retained 10 genes, the core alismatids have retained from six to just one of these genes. Among the taxa surveyed here the two species of *Zostera* are the only ones that have retained just one ribosomal gene (*rps7*). A few genes (*rps1*, *rps3*, *rps12*) occur in most of the taxa, but not in all, suggesting that all the ribosomal protein genes are dispensable. These data are comparable to those from the genus *Silene*, where some species have only one gene left [4, 55, 56], from *Ajuga reptans*, which retains only three genes [56], and from the genus *Viscum*, where zero to six genes are retained [5, 9].

Mapping the loss of genes from the mitogenome on a phylogeny of the Alismatales reveals that nine genes are lost only once either completely or functionally by pseudogene formation (Fig 3). Three genes are lost twice (*rpl10*, *rps7*, *rps12*), but the history of gene loss of the remaining three ribosomal genes within the core alismatids becomes increasingly complicated with the genes being lost five to six times. Given the phylogeny five recurrent losses of *rps11* must be postulated (Fig 3). However, in this case ambiguities concerning resolution of the Alismatales phylogeny should be considered. An alternative resolution provided by plastid data only [23] would reduce the number of losses to three, and another based exclusively on mitochondrial data [22] would reduce it to two. The possibility that only one loss has occurred in Alismatales and that the occurrence of *rps11* in *Scheuchzeria* could be attributable to horizontal gene transfer, is not likely though it cannot be excluded. The *rps11* gene in *Scheuchzeria* is more similar to the *rps11* in *Triantha* than to any other available sequence, and thus consistent with vertical rather than horizontal transfer. The *rps11* gene is lost repeatedly in the monocots and in all core eudicots [7], thus repeated loss in Alismatales may not be surprising. The phylogeny also implies five recurrent losses of *rps1*. All are mapped to terminal branches and none of these postulated losses are affected by the choice among the recently published phylogenies [21–23]. The greatest number of complete or functional losses of ribosomal protein genes is detected in *rps3*, which is lost six times from terminal branches in the phylogeny. Even in this case, the number of invoked losses is unaffected by the choice of phylogeny. Whereas Adams et al. [7] reported multiple losses of *rps1* in the angiosperms they found only few losses of *rps3*, thus the recurrent losses in the core alismatids are surprising.

Whether genes missing from the mitogenome are completely lost or transferred to the nuclear genome is rarely known in detail, but in *Zostera marina* the complete nuclear genome sequence [24] provides evidence that at least four of the ribosomal protein coding genes have been transferred to the nucleus. Three of these genes (*rps2*, *rps4*, *rps14*) are missing from the mitogenome of all core alismatids. These NUMTs in *Zostera* are all very divergent compared to the mitochondrial genes in *Triantha* and *Spirodela* (approximately 40–60% similarity), suggesting ancient transfer events. Thus, *rps14* could have been functionally transferred to the nucleus in a common ancestor of the core alismatids and Araceae, and *rps2* and *rps4* subsequently in the common ancestor of the core alismatids (Fig 3). Each of the transfer events could potentially have occurred previously and include a period of time with co-existence of nuclear and mitochondrial gene copies as found in some other plants (e.g., [8]). The *rps2* gene has possibly been lost from the mitogenome of all eudicots and functionally transferred to the nucleus [7], but even if we now have evidence that it is also functionally transferred in *Zostera* and thus possible so in all core alismatids, we cannot be certain whether this involves a second transfer event or whether some groups (e.g., Araceae and Tofieldiaceae) have retained the mitochondrial gene copy. Functional transfers of *rps14* have occurred in both eudicots and members of the Poales (e.g., [42, 57, 58]), and a functional transfer of *rps4* is found in Geraniaceae [58], thus for these two genes the transfers observed in core alismatids are independent events.

The *rps12* gene, which is only rarely lost from angiosperm mitogenomes [7], is present in most members of the Alismatales except *Zostera* and *Ranalisma*, suggesting that it was either lost/transferred twice, recently, or that a copy was transferred in a common ancestor. In most Alismatales the coding sequence of *rps12* includes one reading frame, whereas the nuclear gene in *Zostera* is split into two exons (scaffold 89, LFYR01002109), the second corresponding to the mitochondrial gene. Expansion of the coding sequence, insertion of an intron and moderate sequence similarity between the nuclear *rps12* second exon and mitochondrial *rps12* sequences (approximately 75% for all Alismatales), suggest that the transfer is not recent. Weak phylogenetic evidence suggests that transfer took place ancestrally in this clade including Maundiaceae, Juncaginaceae, Cymodoceaceae, Potamogetonaceae and Zosteraceae (see Fig 3 for the Alismatales phylogeny).

Although *rps7* is the most frequently lost ribosomal protein gene, according to Adams et al. [7], it appears only rarely to be functionally transferred to the nucleus [58, 59]. The complete gene has been transferred to the nuclear genome of *Zostera* (scaffold 54, LFYR01001529), but it is not likely to be functional. The nuclear *rps7* gene sequence is identical to the gene sequence remaining in the mitogenome and it is included in a >38 kb NUMT that is almost identical to the mitogenome sequence, which strongly suggests a very recent transfer of DNA from the mitogenome to the nucleus.

In addition to the genes annotated as mitochondrial in the *Zostera* nuclear genome, BLASTN searches for the remaining ribosomal protein genes missing from the *Zostera* mitogenome further suggest that *rps1* may also have been transferred. Absence of a mitochondrial *rps1* gene in *Maundia* and a presence of a mitochondrial pseudogene in *Zannichellia* coupled with phylogenetic evidence suggest that a transfer occurred in the common ancestor of the group including Maundiaceae, Cymodoceaceae, Potamogetonaceae and Zosteraceae. Functional transfers of *rps1* are known from several eudicots (e.g., [42, 59]).

Although a BLASTN search for *rpl10* reveals a short sequence to be present in nuclear genome of *Zostera* we do not consider it a likely replacement of the mitochondrial *rpl10* gene. It is possible that the mitochondrial *rpl10* gene has been functionally replaced by a duplicated nuclear encoded chloroplast counterpart as described in other monocots and Brassicaceae [60]. The nuclear genome of *Zostera* does include an *rpl10* gene annotated as encoding a “putative 50S ribosomal protein L10” (LFYR01000749), which appear to have a plastid origin. Other putative ribosomal genes in the *Zostera* nuclear genome appear to have only remote similarity to seed plant mitochondrial genes, thus functional replacement may possibly account for the remaining ribosomal protein genes apparently missing in both the nuclear and mitochondrial genome of *Zostera*.

## Supporting information

S1 Table Reference mitogenome and plastome sequences from GenBank. = indicates that the same species was used.
(DOCX)

S2 Table Sequence reads and coverage. Total number of reads from 454 and Illumina sequencing, number of reads mapped to the mitogenomes and mean coverage of reads.
(DOCX)

S3 Table Gene content in mitogenomes of five representatives of Alismatales. The length of coding sequences in kb. Z = *Zostera*. Ψ = pseudogene or fragment. CP = tRNA gene located in region of plastid origin.
(DOCX)

S4 Table Repeated sequence in *Stratiotes aloides* and *Zostera marina*. (DOCX)

S5 Table Inserts of plastid origin in the mitogenomes of *Stratiotes aloides*, *Butomus umbellatus* and *Zostera marina*. (DOCX)

S6 Table Similarity and phylogenetic relationship of plastid gene fragments located in mitochondrial genomes of *Butomus umbellatus*, *Zostera marina* and *Stratiotes aloides*. BLASTN and phylogenetic analyses are done using matrices from Ross et al. [23] including ca. 150 taxa mostly from the Alismatales.
(DOCX)

S7 Table Sequence similarity between the mitochondrial and nuclear genome of *Zostera marina*. (DOCX)
