# Supplementary material for: Mitochondrial genome evolution in Alismatales: Size reduction and extensive loss of ribosomal protein genes
Source: PLoS One. 2017 May 17;12(5):e0177606. doi: 10.1371/journal.pone.0177606 (PMC5435185; doi:10.1371/journal.pone.0177606)
Supplement: S1 Table — = indicates that the same species was used. (DOCX) [file pone.0177606.s001.docx]

**S1 Table**. **Reference mitogenome and plastome sequences from GenBank.**  = indicates that the same species was used.

| **Higher taxon** | **Species** | **MT**  **Acc. No.** | **Species** | **CP**  **Acc. No.** |
| --- | --- | --- | --- | --- |
| [**Magnoliales**](http://www.ncbi.nlm.nih.gov/Taxonomy/Browser/wwwtax.cgi?mode=Undef&id=3400&lvl=3&keep=1&srchmode=1&unlock) | *Liriodendron tulipifera* | [NC_021152](http://www.ncbi.nlm.nih.gov/nuccore/484759929) | = | NC_008326 |
| **Brassicales** | *Arabidopsis thaliana* | NC_001284 | = | NC_000932 |
| **Brassicales** | *Brassica napus* | NC_008285 | = | NC_016734 |
| **Brassicales** | *Carica papaya* | NC_012116 | = | NC_010323 |
| [**Brassicales**](http://www.ncbi.nlm.nih.gov/Taxonomy/Browser/wwwtax.cgi?mode=Undef&id=3699&lvl=3&keep=1&srchmode=1&unlock) | *Raphanus sativus* | NC_018551 | = | NC_024469 |
| [**Cucurbitales**](http://www.ncbi.nlm.nih.gov/Taxonomy/Browser/wwwtax.cgi?mode=Undef&id=71239&lvl=3&keep=1&srchmode=1&unlock) | *Citrullus lanatus* | NC_014043 | = |  |
| [**Cucurbitales**](http://www.ncbi.nlm.nih.gov/Taxonomy/Browser/wwwtax.cgi?mode=Undef&id=71239&lvl=3&keep=1&srchmode=1&unlock) | *Cucumis sativus* | NC_016005 | = | NC_007144 |
| [**Cucurbitales**](http://www.ncbi.nlm.nih.gov/Taxonomy/Browser/wwwtax.cgi?mode=Undef&id=71239&lvl=3&keep=1&srchmode=1&unlock) | *Cucurbita pepo* | NC_014050 |  |  |
| [**Fabales**](http://www.ncbi.nlm.nih.gov/Taxonomy/Browser/wwwtax.cgi?mode=Undef&id=72025&lvl=3&keep=1&srchmode=1&unlock) | *Glycine max* | NC_020455 | = | NC_007942 |
| [**Fabales**](http://www.ncbi.nlm.nih.gov/Taxonomy/Browser/wwwtax.cgi?mode=Undef&id=72025&lvl=3&keep=1&srchmode=1&unlock) | *Lotus japonicus* | NC_016743 | = | NC_002694 |
| [**Fabales**](http://www.ncbi.nlm.nih.gov/Taxonomy/Browser/wwwtax.cgi?mode=Undef&id=72025&lvl=3&keep=1&srchmode=1&unlock) | *Millettia pinnata* | NC_016742 | = | NC_016708 |
| [**Fabales**](http://www.ncbi.nlm.nih.gov/Taxonomy/Browser/wwwtax.cgi?mode=Undef&id=72025&lvl=3&keep=1&srchmode=1&unlock) | *Vigna radiata* | NC_015121 | = | NC_013843 |
| [**Malpighiales**](http://www.ncbi.nlm.nih.gov/Taxonomy/Browser/wwwtax.cgi?mode=Undef&id=3646&lvl=3&keep=1&srchmode=1&unlock) | *Ricinus communis* | NC_015141 | = | NC_016736 |
| [**Rosales**](http://www.ncbi.nlm.nih.gov/Taxonomy/Browser/wwwtax.cgi?mode=Undef&id=3744&lvl=3&keep=1&srchmode=1&unlock) | *Malus x domestica* | NC_018554 |  |  |
| [**Vitales**](http://www.ncbi.nlm.nih.gov/Taxonomy/Browser/wwwtax.cgi?mode=Undef&id=403667&lvl=3&keep=1&srchmode=1&unlock) | *Vitis vinifera* | NC_012119 | = | NC_007957 |
| [**Caryophyllales**](http://www.ncbi.nlm.nih.gov/Taxonomy/Browser/wwwtax.cgi?mode=Undef&id=3524&lvl=3&keep=1&srchmode=1&unlock) | *Beta vulgaris* | NC_002511 |  |  |
| [**Caryophyllales**](http://www.ncbi.nlm.nih.gov/Taxonomy/Browser/wwwtax.cgi?mode=Undef&id=3524&lvl=3&keep=1&srchmode=1&unlock) | *Silene latifolia* | NC_014487 | = | NC_016730 |
| [**Gentianales**](http://www.ncbi.nlm.nih.gov/Taxonomy/Browser/wwwtax.cgi?mode=Undef&id=4055&lvl=3&keep=1&srchmode=1&unlock) | *Asclepias syriaca* | NC_022796 | = | NC_022432 |
| [**Lamiales**](http://www.ncbi.nlm.nih.gov/Taxonomy/Browser/wwwtax.cgi?mode=Undef&id=4143&lvl=3&keep=1&srchmode=1&unlock) | *Boea hygrometrica* | NC_016741 |  |  |
| [**Lamiales**](http://www.ncbi.nlm.nih.gov/Taxonomy/Browser/wwwtax.cgi?mode=Undef&id=4143&lvl=3&keep=1&srchmode=1&unlock) | *Erythranthe guttata* | NC_018041 | *E. lutea* | NC_030212 |
| [**Solanales**](http://www.ncbi.nlm.nih.gov/Taxonomy/Browser/wwwtax.cgi?mode=Undef&id=4069&lvl=3&keep=1&srchmode=1&unlock) | *Nicotiana tabacum* | NC_006581 | = | NC_001879 |
| [**Apiales**](http://www.ncbi.nlm.nih.gov/Taxonomy/Browser/wwwtax.cgi?mode=Undef&id=4036&lvl=3&keep=1&srchmode=1&unlock) | *Daucus carota* | NC_017855 | = | NC_008325 |
| [**Arecales**](http://www.ncbi.nlm.nih.gov/Taxonomy/Browser/wwwtax.cgi?mode=Undef&id=40551&lvl=3&keep=1&srchmode=1&unlock) | *Phoenix dactylifera* | NC_016740 | = | NC_013991 |
| [**Poales**](http://www.ncbi.nlm.nih.gov/Taxonomy/Browser/wwwtax.cgi?mode=Undef&id=38820&lvl=3&keep=1&srchmode=1&unlock) | *Oryza sativa* | NC_007886 | = | NC_008155 |
| [**Poales**](http://www.ncbi.nlm.nih.gov/Taxonomy/Browser/wwwtax.cgi?mode=Undef&id=38820&lvl=3&keep=1&srchmode=1&unlock) | *Sorghum bicolor* | NC_008360 | = | NC_008602 |
| **Alismatales** | *Spirodela polyrhiza* | NC_017840 | = | NC_015891 |
| **Alismatales** | *Butomus umbellatus* | NC_021399 |  |  |
| **Alismatales** | *Elodea canadensis* |  |  | NC_018541 |
| **Alismatales** | *Lemna minor* |  |  | NC_010109 |

Mitochondrial ribosomal protein genes were always extracted from: NC_007579, NC_007982, NC_008331, NC_008333, NC_008362, NC_010303, NC_013816, NC_015994, NC_016118, NC_016120, NC_016123, NC_021092, NC_022666, NC_022714, NC_023103, NC_023209, NC_023337, NC_023338, NC_024293, NC_024429, NC_024624, NC_026515, AP014526, JX065074
