# Supplementary material for: Mitochondrial genome evolution in Alismatales: Size reduction and extensive loss of ribosomal protein genes
Source: PLoS One. 2017 May 17;12(5):e0177606. doi: 10.1371/journal.pone.0177606 (PMC5435185; doi:10.1371/journal.pone.0177606)
Supplement: S2 Table — Total number of reads from 454 and Illumina sequencing, number of reads mapped to the mitogenomes and mean coverage of reads. (DOCX) [file pone.0177606.s002.docx]

**S2 Table**. **Sequence reads and coverage.** Total number of reads from 454 and Illumina sequencing, number of reads mapped to the mitogenomes and mean coverage of reads.

Reads, total Reads, mapped ×-coverage (mean)

Species 454 Illumina 454 Illumina 454 Illumina

*Stratiotes aloides* 60,812 7,955,016 5,190 799,815 5.2 223.9

*Zostera marina* 167,419 13,857,242 38,263 388,351 71.6 210.1
