# Supplementary material for: Mitochondrial genome evolution in Alismatales: Size reduction and extensive loss of ribosomal protein genes
Source: PLoS One. 2017 May 17;12(5):e0177606. doi: 10.1371/journal.pone.0177606 (PMC5435185; doi:10.1371/journal.pone.0177606)
Supplement: S3 Table — The length of coding sequences in kb. Z = Zostera. Ψ = pseudogene or fragment. CP = tRNA gene located in region of plastid origin. (DOCX) [file pone.0177606.s003.docx]

**S3 Table**. **Gene content in mitogenomes of five representatives of Alismatales**. The length of coding sequences in bp. Z = *Zostera*. Ψ = pseudogene or fragment. CP = tRNA gene located in region of plastid origin.

Gene *Spirodela Butomus Stratiotes Z. marina Z. noltii* Notes

atp1 1524 1470 1464 1560 1560

atp4 531 606 594 552 576

atp6 720 915 876 744 771

atp8 471 480 480 585 720

atp9 225 225 225 225 225

ccmB 612 621 621 615 621

ccmC 783 780 783 723 717

ccmFCexon1 759 767 758 773 782

exon2 558 613 604 631 631

ccmFN 1716 1314 1791 2253 2076

cob 1182 1179 1179 1170 1206

cox1 1584 1584 1584 1572 1572

cox2exon1 390 715* 706* 742* 748* *no intron cox2i373

exon2 316 - - - -

exon3 77* 83* 98 86 86 *not annotated

cox3 798 798 798 798 798

matR 1962 1962 2001 2784 2586

mttB 771 858 741 750 750

nad1 exon1 372 385 385 385 385

exon2 81 83 83 83 83

exon3 195 192 192 192 192

exon4 59* 59 59 59 59 * not annotated

exon5 259 259 259 259 259

nad2 exon1 153 153 153 153 153

exon2 351 392 392 392 392

exon3 156 161 161 161 161

exon4 582 573 573 573 573

exon5 189 188 191 188 188

nad3 357 357 357 357 357

nad4 exon1 459 461 461 461 461

exon2 513 515 515 515 515

exon3 420 423 423 423 423

exon4 90 89 89 89 89

nad4L 303 303 303 303 303

nad5 exon1 228 231 231 231 231

exon2 1215 1215 1215 1215 1215

exon3 21* 21 21 21 21 *not annotated

exon4 396 393 393 393 393

exon5 150 150 150 156 156

nad6 660 690 660 633 633

nad7 exon1 156 143 143 134 134

exon2 69* 69 69 69 69 *not annotated

exon3 465 467 467 467 467

exon4 246 244 244 244 244

exon5 261 256 256 256 256

nad9 573 618 585 585 585

rpl5 555 - - - -

rpl10 474 489* 459 - - *not annotated

rpl16 540 ψ(398)* ψ (454) - - *not annotated

rps1 519 558 504 - -

rps2 660 - - - -

rps3 exon1 74* 74 74 - - *not annotated

exon2 1603* 1516 1498 ψ(403) ψ(160+408) *length adjusted

rps4 1050 - - - -

rps7 390 432 ψ (493) 396 396

rps12 378 378 378 - -

rps13 351 - - - -

rps14 ψ(259) - - - -

rps19 ψ(289) - - - -

sdh3 - - - - -

sdh4 453 - - - -

rrn5 119 123 113 124 120

rrn18 1942 1876 2126 1851 1842

rrn26 2619 3076 3178 3248 3210

trnC(gca) 71 71 71 71

trnD(guc) 74 74 74 74

trnE(uuc) 72 72 72 72

trnF(gaa) 74 - - -

trnF(gcu) 88 - - -

trnG(gcc) 72 - - -

trnH(gug) 74 74+75 74 -

trnI(cau) 81 CP CP -

trnK(cuu) - 72 - -

trnK(uuu) CP CP - -

trnfM(cau) 74 74 74 74+74

trnM(cau) 73 74 74 81

trnN(guu) CP - -

trnP(ugg) CP - CP -

trnQ(uug) 72 72 72 72

trnS(gga) 87 - - -

trnS(gcu) CP - - -

trnS(uga) 87 - - -

trnW(cca) 74 CP CP CP

trnY(gua) 83 83 83 CP
