# Supplementary material for: Mitochondrial genome evolution in Alismatales: Size reduction and extensive loss of ribosomal protein genes
Source: PLoS One. 2017 May 17;12(5):e0177606. doi: 10.1371/journal.pone.0177606 (PMC5435185; doi:10.1371/journal.pone.0177606)
Supplement: S4 Table — (DOCX) [file pone.0177606.s004.docx]

**S4 Table. Repeated sequences in *Stratiotes aloides* and *Zostera marina***

*Stratiotes:*

| **Start** | **End** | **Start** | **End** | **Expectation** | **Similarity (%)** | **Length(bp)** | |
| --- | --- | --- | --- | --- | --- | --- | --- |
| 1 | 382 | 79584 | 79965 | 0.0 | 100.000 | | 382 |
| 14075 | 14192 | 110065 | 109948 | 1.42e-55 | 100.000 | | 118 |
| 31479 | 31632 | 31633 | 31786 | 1.38e-75 | 100.000 | | 154 |
| 79483 | 79583 | 97050 | 96950 | 4.01e-46 | 100.000 | | 101 |
| 79483 | 79583 | 348958 | 349058 | 4.01e-46 | 100.000 | | 101 |
| 80362 | 80521 | 237458 | 237299 | 1.38e-75 | 98.750 | | 160 |
| 96950 | 97058 | 349058 | 348950 | 1.43e-50 | 100.000 | | 109 |
| 108707 | 108809 | 289220 | 289117 | 6.75e-39 | 95.192 | | 104 |
| 120025 | 120126 | 155528 | 155629 | 1.11e-46 | 100.000 | | 102 |
| 120246 | 120399 | 177573 | 177726 | 1.38e-75 | 100.000 | | 154 |
| 156591 | 156728 | 219106 | 219243 | 1.09e-61 | 97.826 | | 138 |
| 163870 | 164041 | 164042 | 164213 | 1.36e-85 | 100.000 | | 172 |
| 251372 | 251612 | 262533 | 262293 | 5.99e-124 | 100.000 | | 241 |

*Zostera:*

| **Start** | **End** | **Start** | **End** | **Expectation** | **Similarity (%)** | **Length(bp)** |
| --- | --- | --- | --- | --- | --- | --- |
| 1 | 1288 | 97324 | 98611 | 0.0 | 100.000 | 1288 |
| 1 | 263 | 13234 | 13494 | 1.92e-79 | 87.313 | 268 |
| 1 | 263 | 178475 | 178736 | 1.87e-99 | 91.791 | 268 |
| 1 | 263 | 83412 | 83673 | 5.23e-95 | 90.299 | 268 |
| 1 | 263 | 178505 | 178766 | 1.84e-109 | 93.962 | 265 |
| 1 | 263 | 83442 | 83703 | 5.15e-105 | 92.453 | 265 |
| 1 | 264 | 13263 | 13525 | 5.01e-125 | 97.348 | 264 |
| 1 | 237 | 83472 | 83707 | 5.45e-65 | 86.066 | 244 |
| 1 | 237 | 178535 | 178770 | 5.45e-65 | 86.066 | 244 |
| 1 | 185 | 13204 | 13387 | 1.98e-59 | 89.305 | 187 |
| 1 | 185 | 178416 | 178600 | 1.17e-66 | 91.444 | 187 |
| 1 | 185 | 178446 | 178630 | 1.17e-66 | 91.444 | 187 |
| 1 | 185 | 83382 | 83567 | 5.38e-75 | 93.583 | 187 |
| 1 | 185 | 83352 | 83537 | 7.01e-69 | 91.444 | 187 |
| 1 | 185 | 83323 | 83507 | 1.50e-75 | 93.548 | 186 |
| 1 | 155 | 137135 | 136974 | 2.67e-28 | 81.707 | 164 |
| 22 | 185 | 83314 | 83478 | 1.18e-61 | 92.169 | 166 |
| 22 | 185 | 13195 | 13357 | 9.00e-73 | 96.951 | 164 |
| 22 | 185 | 178407 | 178570 | 5.34e-80 | 99.390 | 164 |
| 52 | 185 | 97281 | 97419 | 9.26e-53 | 93.525 | 139 |
| 55 | 185 | 97318 | 97449 | 2.61e-43 | 90.977 | 133 |
| 133 | 237 | 5830 | 5725 | 3.52e-12 | 80.180 | 111 |
| 152 | 263 | 5840 | 5729 | 9.26e-53 | 100.000 | 112 |
| 407 | 511 | 1074 | 1172 | 3.45e-27 | 88.571 | 105 |
| 1290 | 1450 | 141193 | 141033 | 2.48e-78 | 99.379 | 161 |
| 5717 | 5830 | 83715 | 83602 | 7.16e-54 | 100.000 | 114 |
| 5717 | 5830 | 178778 | 178665 | 7.16e-54 | 100.000 | 114 |
| 5725 | 5843 | 83677 | 83560 | 9.73e-18 | 81.452 | 124 |
| 5725 | 5843 | 178740 | 178623 | 9.73e-18 | 81.452 | 124 |
| 5725 | 5843 | 13527 | 13410 | 9.32e-48 | 95.798 | 119 |
| 5729 | 5843 | 97586 | 97472 | 9.26e-53 | 99.130 | 115 |
| 5808 | 5934 | 45116 | 44981 | 5.81e-20 | 81.159 | 138 |
| 7517 | 7653 | 49208 | 49059 | 2.61e-43 | 89.333 | 150 |
| 8892 | 9010 | 31884 | 31766 | 5.53e-55 | 99.160 | 119 |
| 9733 | 9853 | 21336 | 21217 | 1.54e-55 | 99.174 | 121 |
| 9751 | 9855 | 52833 | 52937 | 3.35e-47 | 99.048 | 105 |
| 9754 | 9855 | 51088 | 50987 | 3.35e-47 | 100.000 | 102 |
| 12304 | 12467 | 137020 | 136857 | 5.34e-80 | 99.390 | 164 |
| 12304 | 12416 | 41969 | 41858 | 2.09e-19 | 82.906 | 117 |
| 12316 | 12418 | 54177 | 54077 | 1.60e-25 | 87.379 | 103 |
| 12316 | 12418 | 92463 | 92363 | 3.47e-22 | 85.437 | 103 |
| 12316 | 12418 | 187526 | 187426 | 3.47e-22 | 85.437 | 103 |
| 12317 | 12432 | 51149 | 51038 | 1.60e-25 | 85.470 | 117 |
| 13186 | 13528 | 178398 | 178741 | 3.79e-141 | 93.143 | 350 |
| 13186 | 13494 | 97272 | 97586 | 6.81e-89 | 85.714 | 322 |
| 13186 | 13447 | 83305 | 83568 | 2.41e-98 | 90.977 | 266 |
| 13186 | 13320 | 164662 | 164801 | 5.69e-35 | 86.525 | 141 |
| 13195 | 13528 | 83343 | 83678 | 4.97e-130 | 91.520 | 342 |
| 13195 | 13527 | 178437 | 178770 | 6.39e-134 | 92.582 | 337 |
| 13195 | 13527 | 83373 | 83707 | 1.06e-136 | 92.582 | 337 |
| 13195 | 13402 | 13313 | 13509 | 4.31e-51 | 85.308 | 211 |
| 13195 | 13393 | 13254 | 13452 | 9.00e-73 | 91.542 | 201 |
| 13196 | 13525 | 97257 | 97587 | 1.37e-135 | 92.857 | 336 |
| 13196 | 13452 | 83290 | 83543 | 4.10e-86 | 88.846 | 260 |
| 13196 | 13349 | 164640 | 164801 | 2.69e-23 | 79.755 | 163 |
| 13226 | 13443 | 137143 | 136913 | 2.63e-38 | 80.258 | 233 |
| 13284 | 13509 | 83314 | 83552 | 1.95e-69 | 87.137 | 241 |
| 14120 | 14229 | 21076 | 21189 | 4.49e-21 | 83.761 | 117 |
| 14120 | 14229 | 53076 | 52963 | 4.49e-21 | 83.761 | 117 |
| 14174 | 14289 | 76838 | 76728 | 2.61e-43 | 94.828 | 116 |
| 14204 | 14317 | 16250 | 16144 | 2.70e-18 | 82.906 | 117 |
| 14204 | 14308 | 45347 | 45249 | 7.57e-14 | 81.481 | 108 |
| 16073 | 16286 | 102935 | 103142 | 2.05e-34 | 80.000 | 220 |
| 16152 | 16255 | 45249 | 45352 | 2.61e-43 | 97.115 | 104 |
| 17414 | 17614 | 32550 | 32757 | 6.91e-79 | 92.788 | 208 |
| 17414 | 17614 | 115033 | 115240 | 6.91e-79 | 92.788 | 208 |
| 19730 | 19873 | 109099 | 108956 | 7.01e-69 | 99.306 | 144 |
| 20807 | 20956 | 119033 | 118884 | 3.28e-62 | 95.333 | 150 |
| 20938 | 21078 | 33571 | 33715 | 1.21e-41 | 88.435 | 147 |
| 20938 | 21078 | 116054 | 116198 | 1.21e-41 | 88.435 | 147 |
| 20955 | 21062 | 162761 | 162653 | 7.26e-44 | 96.330 | 109 |
| 21074 | 21183 | 148682 | 148789 | 2.02e-44 | 96.364 | 110 |
| 22741 | 23746 | 171071 | 170067 | 0.0 | 99.901 | 1006 |
| 23152 | 23281 | 39358 | 39229 | 9.13e-63 | 100.000 | 130 |
| 26243 | 26345 | 163098 | 162997 | 7.26e-44 | 98.058 | 103 |
| 26246 | 26398 | 45116 | 44977 | 5.65e-40 | 87.582 | 153 |
| 26264 | 26394 | 103164 | 103034 | 5.61e-45 | 91.729 | 133 |
| 26275 | 26394 | 189772 | 189899 | 4.46e-26 | 84.615 | 130 |
| 26286 | 26617 | 56501 | 56844 | 2.91e-147 | 94.477 | 344 |
| 26290 | 26394 | 76361 | 76258 | 2.65e-33 | 91.509 | 106 |
| 26295 | 26411 | 135110 | 135226 | 1.57e-40 | 92.373 | 118 |
| 26295 | 26398 | 162990 | 162887 | 5.65e-40 | 95.192 | 104 |
| 26590 | 26946 | 41923 | 42279 | 1.67e-179 | 98.319 | 357 |
| 28901 | 29145 | 191237 | 191481 | 1.08e-126 | 100.000 | 245 |
| 29712 | 29833 | 166843 | 166722 | 2.56e-58 | 100.000 | 122 |
| 32324 | 36019 | 114807 | 118502 | 0.0 | 100.000 | 3696 |
| 33228 | 33531 | 93800 | 94107 | 3.08e-107 | 89.809 | 314 |
| 33491 | 33640 | 138977 | 138827 | 1.52e-65 | 96.711 | 152 |
| 33498 | 33721 | 83950 | 83733 | 5.26e-90 | 93.750 | 224 |
| 33498 | 33721 | 179013 | 178796 | 5.26e-90 | 93.750 | 224 |
| 33509 | 33714 | 138835 | 138636 | 1.15e-81 | 93.689 | 206 |
| 39096 | 39238 | 121802 | 121944 | 2.52e-68 | 99.301 | 143 |
| 39229 | 39358 | 170532 | 170661 | 9.13e-63 | 100.000 | 130 |
| 41846 | 41958 | 54066 | 54178 | 1.25e-21 | 83.621 | 116 |
| 41846 | 41958 | 92352 | 92464 | 1.25e-21 | 83.621 | 116 |
| 41846 | 41958 | 187415 | 187527 | 1.25e-21 | 83.621 | 116 |
| 41846 | 41943 | 52951 | 52850 | 2.11e-14 | 82.075 | 106 |
| 41858 | 41969 | 136908 | 137020 | 9.73e-18 | 82.051 | 117 |
| 41858 | 41968 | 50988 | 51098 | 3.50e-17 | 81.897 | 116 |
| 43915 | 44089 | 50779 | 50953 | 1.91e-84 | 98.857 | 175 |
| 43941 | 44050 | 80718 | 80823 | 3.47e-22 | 84.821 | 112 |
| 44966 | 45071 | 135224 | 135119 | 2.00e-49 | 100.000 | 106 |
| 44970 | 45071 | 162879 | 162981 | 1.56e-45 | 99.029 | 103 |
| 49150 | 49347 | 57378 | 57577 | 8.75e-93 | 97.500 | 200 |
| 49158 | 49287 | 163066 | 162940 | 1.21e-41 | 90.840 | 131 |
| 49181 | 49287 | 135057 | 135160 | 1.59e-30 | 89.815 | 108 |
| 50805 | 50914 | 80718 | 80823 | 3.47e-22 | 84.821 | 112 |
| 50961 | 51095 | 136873 | 137016 | 4.37e-41 | 88.889 | 144 |
| 50969 | 51074 | 54059 | 54163 | 3.47e-22 | 85.047 | 107 |
| 50987 | 51144 | 140271 | 140099 | 7.36e-34 | 83.237 | 173 |
| 50987 | 51095 | 52937 | 52829 | 9.32e-48 | 98.165 | 109 |
| 51038 | 51149 | 136892 | 137007 | 1.60e-25 | 85.470 | 117 |
| 51081 | 51191 | 52905 | 52793 | 2.05e-34 | 90.351 | 114 |
| 52799 | 52952 | 92506 | 92351 | 2.69e-23 | 80.000 | 160 |
| 52799 | 52952 | 187569 | 187414 | 2.69e-23 | 80.000 | 160 |
| 52830 | 52952 | 54184 | 54065 | 2.08e-24 | 83.871 | 124 |
| 52836 | 53197 | 21316 | 20956 | 0.0 | 99.448 | 362 |
| 52969 | 53078 | 148789 | 148682 | 2.02e-44 | 96.364 | 110 |
| 53090 | 53197 | 162653 | 162760 | 3.38e-42 | 95.413 | 109 |
| 53109 | 53212 | 71736 | 71839 | 2.61e-43 | 97.115 | 104 |
| 54047 | 54208 | 92332 | 92494 | 6.96e-74 | 97.546 | 163 |
| 54047 | 54208 | 187395 | 187557 | 6.96e-74 | 97.546 | 163 |
| 54065 | 54170 | 156566 | 156674 | 2.69e-23 | 85.714 | 112 |
| 54077 | 54177 | 136906 | 137008 | 1.60e-25 | 87.379 | 103 |
| 56456 | 56608 | 135068 | 135209 | 7.31e-39 | 87.179 | 156 |
| 56500 | 56640 | 103142 | 103002 | 1.55e-50 | 92.199 | 141 |
| 56503 | 56621 | 76363 | 76245 | 5.53e-55 | 99.160 | 119 |
| 56510 | 56608 | 162990 | 162891 | 7.31e-39 | 96.040 | 101 |
| 57385 | 57517 | 163067 | 162940 | 9.45e-38 | 88.806 | 134 |
| 57411 | 57517 | 135057 | 135160 | 1.59e-30 | 89.815 | 108 |
| 59510 | 59612 | 76710 | 76602 | 2.03e-39 | 94.495 | 109 |
| 59526 | 59640 | 71888 | 71775 | 1.57e-40 | 93.043 | 115 |
| 59557 | 59648 | 83963 | 83861 | 9.80e-13 | 81.553 | 103 |
| 59557 | 59648 | 179026 | 178924 | 9.80e-13 | 81.553 | 103 |
| 59648 | 60187 | 127712 | 127186 | 1.03e-156 | 85.609 | 542 |
| 60176 | 60665 | 99158 | 99638 | 2.40e-103 | 80.972 | 494 |
| 61105 | 61210 | 189910 | 190010 | 2.06e-29 | 89.720 | 107 |
| 71736 | 71869 | 162673 | 162805 | 2.03e-39 | 89.209 | 139 |
| 71787 | 71910 | 76588 | 76709 | 1.54e-55 | 98.387 | 124 |
| 73552 | 73718 | 160468 | 160302 | 2.47e-83 | 100.000 | 167 |
| 76245 | 76363 | 103021 | 103139 | 2.59e-48 | 95.798 | 119 |
| 76258 | 76356 | 135209 | 135110 | 7.31e-39 | 96.040 | 101 |
| 76258 | 76356 | 162891 | 162990 | 7.31e-39 | 96.040 | 101 |
| 76425 | 76547 | 92599 | 92466 | 2.70e-18 | 80.741 | 135 |
| 76425 | 76547 | 187662 | 187529 | 2.70e-18 | 80.741 | 135 |
| 76454 | 76588 | 189917 | 189783 | 1.52e-65 | 100.000 | 135 |
| 76611 | 76800 | 162779 | 162951 | 4.37e-41 | 84.375 | 192 |
| 80237 | 80486 | 176349 | 176100 | 1.79e-129 | 100.000 | 250 |
| 82815 | 83537 | 96782 | 97508 | 0.0 | 96.708 | 729 |
| 83290 | 83776 | 178408 | 178875 | 3.93e-116 | 83.068 | 502 |
| 83297 | 83410 | 164655 | 164769 | 7.52e-19 | 82.203 | 118 |
| 83314 | 83776 | 83373 | 83812 | 6.67e-104 | 82.068 | 474 |
| 83315 | 83475 | 137081 | 136913 | 3.45e-27 | 80.117 | 171 |
| 83343 | 93834 | 178407 | 188897 | 0.0 | 99.895 | 10492 |
| 83374 | 83499 | 164640 | 164769 | 2.67e-28 | 83.969 | 131 |
| 83376 | 83567 | 97318 | 97508 | 2.48e-78 | 93.782 | 193 |
| 83404 | 83622 | 137143 | 136913 | 1.57e-40 | 80.342 | 234 |
| 83406 | 83673 | 97318 | 97586 | 2.41e-98 | 90.511 | 274 |
| 83436 | 83703 | 97318 | 97586 | 2.38e-108 | 92.620 | 271 |
| 83463 | 83625 | 97281 | 97449 | 9.06e-68 | 94.083 | 169 |
| 83466 | 83707 | 97318 | 97560 | 2.52e-68 | 86.345 | 249 |
| 83714 | 83939 | 138646 | 138897 | 1.21e-46 | 81.890 | 254 |
| 83733 | 83950 | 116204 | 115981 | 5.26e-90 | 93.750 | 224 |
| 83740 | 83939 | 138636 | 138835 | 1.12e-101 | 100.000 | 200 |
| 83813 | 83950 | 138827 | 138970 | 1.56e-45 | 90.345 | 145 |
| 92351 | 92456 | 156566 | 156674 | 1.25e-21 | 84.821 | 112 |
| 92363 | 92463 | 136906 | 137008 | 3.47e-22 | 85.437 | 103 |
| 92406 | 92512 | 92920 | 93035 | 1.63e-15 | 81.034 | 116 |
| 92470 | 92585 | 162854 | 162739 | 5.53e-55 | 100.000 | 116 |
| 92570 | 92793 | 94074 | 94297 | 5.08e-115 | 100.000 | 224 |
| 92570 | 92729 | 108002 | 107836 | 4.46e-26 | 80.702 | 171 |
| 93800 | 94107 | 115711 | 116014 | 3.08e-107 | 89.809 | 314 |
| 94071 | 94233 | 108005 | 107836 | 9.59e-28 | 81.034 | 174 |
| 94074 | 94297 | 187633 | 187856 | 5.08e-115 | 100.000 | 224 |
| 97257 | 97586 | 178408 | 178736 | 5.12e-110 | 88.529 | 340 |
| 97269 | 97508 | 178395 | 178630 | 8.93e-78 | 88.477 | 243 |
| 97281 | 97419 | 97375 | 97508 | 9.26e-53 | 93.525 | 139 |
| 97318 | 97586 | 178499 | 178766 | 8.50e-113 | 94.096 | 271 |
| 97318 | 97560 | 178529 | 178770 | 2.52e-68 | 86.400 | 250 |
| 97318 | 97449 | 97378 | 97508 | 2.61e-43 | 90.977 | 133 |
| 97320 | 97478 | 137139 | 136974 | 1.59e-30 | 82.143 | 168 |
| 97730 | 97834 | 98397 | 98495 | 3.45e-27 | 88.571 | 105 |
| 100128 | 101141 | 127183 | 126161 | 0.0 | 81.395 | 1032 |
| 103034 | 103249 | 162891 | 163098 | 5.65e-40 | 81.279 | 219 |
| 103034 | 103132 | 135209 | 135110 | 1.59e-30 | 91.089 | 101 |
| 103114 | 103249 | 148861 | 148729 | 5.61e-45 | 91.176 | 136 |
| 103128 | 103235 | 148910 | 148805 | 5.65e-40 | 94.444 | 108 |
| 104098 | 104229 | 106084 | 105962 | 3.42e-32 | 85.606 | 132 |
| 107836 | 108002 | 187792 | 187633 | 4.46e-26 | 80.702 | 171 |
| 115974 | 116123 | 138977 | 138827 | 1.52e-65 | 96.711 | 152 |
| 115981 | 116204 | 179013 | 178796 | 5.26e-90 | 93.750 | 224 |
| 115992 | 116197 | 138835 | 138636 | 1.15e-81 | 93.689 | 206 |
| 122588 | 122754 | 177545 | 177712 | 5.42e-70 | 95.238 | 168 |
| 123194 | 123448 | 149180 | 148926 | 6.44e-129 | 99.216 | 255 |
| 135057 | 135220 | 163043 | 162879 | 5.34e-80 | 99.394 | 165 |
| 136906 | 137008 | 187426 | 187526 | 3.47e-22 | 85.437 | 103 |
| 136913 | 137143 | 178685 | 178467 | 7.31e-39 | 80.342 | 234 |
| 136981 | 137081 | 164769 | 164672 | 7.52e-19 | 84.314 | 102 |
| 137001 | 137143 | 164814 | 164672 | 5.42e-70 | 100.000 | 143 |
| 138636 | 138835 | 178803 | 179002 | 1.12e-101 | 100.000 | 200 |
| 138646 | 138897 | 178777 | 179002 | 1.21e-46 | 81.818 | 253 |
| 138709 | 138897 | 138765 | 138959 | 6.96e-74 | 92.857 | 196 |
| 138827 | 138970 | 178876 | 179013 | 1.56e-45 | 90.278 | 144 |
| 148696 | 148799 | 163132 | 163028 | 5.65e-40 | 95.238 | 105 |
| 155668 | 155804 | 155700 | 155836 | 1.17e-66 | 100.000 | 137 |
| 156566 | 156674 | 187414 | 187519 | 1.25e-21 | 84.404 | 109 |
| 162739 | 162854 | 187648 | 187533 | 5.53e-55 | 100.000 | 116 |
| 164657 | 164769 | 178393 | 178503 | 9.52e-33 | 89.474 | 114 |
| 187469 | 187575 | 187983 | 188098 | 1.63e-15 | 81.034 | 116 |
