# Supplementary material for: Mitochondrial genome evolution in Alismatales: Size reduction and extensive loss of ribosomal protein genes
Source: PLoS One. 2017 May 17;12(5):e0177606. doi: 10.1371/journal.pone.0177606 (PMC5435185; doi:10.1371/journal.pone.0177606)
Supplement: S5 Table — (DOCX) [file pone.0177606.s005.docx]

**S5 Table. Inserts of plastid origin in the mitogenomes of *Stratiotes aloides*, *Butomus umbellatus* and *Zostera marina***

| Location in *Stratiotes* MT | Length | Location in *Elodea* CP | Length | Identified CP genes in MT sequence |
| --- | --- | --- | --- | --- |
| 309,292-321,544 | 12,253 | 10,278-22,598 | 12,321 | *trnR*(partial), *atpA*, *atpF*, *atpH*, *atpI*, *rps2*, *rpoC2*, *rpoC1*(partial) |
| 287,152-295,349 | 8,198 | 61,566-73,670 | 12,015 | *ycf4*(partial), *psbJ*, *psbL*, *psbF*, *psbE*, *petL*, *petG*, *trnW*, *trnP*, *rps12*ex.3,*clpP* |
| 202,251-209,372 | 7,122 | 38,802-45,790 | 6,989 | *psaB*(partial), *psaA*, *ycf3* |
| 61,095-67,531 | 6,437 | 77,156-83,597 | 6,442 | *petB*(partial), *petD*, *rpoA*, *rps11*, *rpl36*, *infA*, *rps8*, *rpl16*(partial) |
| 127,693-133,443 | 5,751 | 104,311-110,178^a^ IR | 6,442 | *trnI*(partial), *trnA*, *rrn23*, *rrn4.5*, *rrn5*, *trnR* |
| 102,147-106,549 | 4,403 | 375-4,717 | 4,343 | *psbA*(partial), *trnK*, *matK* |
| 142,877-145,723 | 2,847 | 99,662-102,102 IR | 2,441 | *rps12*exon2(partial), *trnV*, *rrn16*(partial) |
| 75,177-76,107 | 931 | 98,665-97,922 IR | 744 | *ndhB*(partial) |
| 336,675-337,483 | 809 | 103,577-104,356^a^ IR | 780 | *trnI*(partial) |
| 36,685-37,325 | 641 | 50,633-51,201 | 569 | *ndhK*(partial) |
| Total | 49,392 |  | 50,306 (50,261 excl. overlap) | |

| Location in *Butomus* MT | Length | Location in *Elodea* CP | Length | Identified CP genes in MT sequence |
| --- | --- | --- | --- | --- |
| 368,045-372,941 | 4,897 | 101,863-106,945 IR | 5,719 | *rrn16*, *trnA,* *trnI*, *rrn23*(partial) |
| 47,701-48,603 | 903 | 50,488-51,563 | 1,076 | *ndhK*, *ndhC*(partial) |
| 438,532-438,932 | 401 | 68,360-67,898 | 463 | *trnK*, *trnW* |
| 171,200-171,359 | 160 | 34,467-34,637 | 171 | *psbD*(partial) |
| 215,497-215,653 | 157 | 25,232-25,065 | 156 | *rpoB*(partial) |
| 34,784-34,889 | 106 | 96,097-96,003 IR | 95 |  |
| 299,456-299,540 | 85^b^ | 78,866-78,949 | 85^b^ | *petD*(partial) |
| 62,015-62,099 | 85^b^ | 78,866-78,949 | 85^b^ | *petD*(partial) |
| 249,922-249,992 | 71 | 34,685-34,759 | 75 | *psbD*(partial) |
| 434,652-434,714 | 63 | 84,544-84,488 | 57 | *rps3*(partial) |
| Total | 6,928 |  | 7,982 (7,897 excl. repeats) | |

| Location in *Zostera* MT | Length | Location in *Elodea* CP | Length | Identified CP genes in MT sequence |
| --- | --- | --- | --- | --- |
| 84,033-90,646 | 6,614 | 12,487-14,673^c^ + 53,834-58,064^d^ | 6,418 | *atpF*(partial*), atpH* + *atpE*, *atpB*, *rbcL* |
| 181,496-185,710 | 4,215 | 53,835-58,064^d^ | 4,230 | *atpE*, *atpB*, *rbcL* |
| 122,457-126,125 | 3,669 | 105,395-109,296^e^ IR | 3,902 | *trnA*(partial), *rrn23*, *rrn4.5*, *rrn5* |
| 144,734-148,256 | 3,523 | 90,281-92,325 IR + 113,743-115,310 | 3,613 | *ycf2*(partial) + *ndhF*(partial) |
| 142,008-144,414 | 2,407 | 19,553-22,014 | 2,461 | *rpoC2*(partial), *rpoC1*(partial) |
| 179,097-181,458 | 2,362 | 12,488-14,673^c^ | 2,186 | *atpF*(partial), *atpH* |
| 37,240-39,105 | 1,866 | 34,352-36,331 | 1,980 | *psbD*(partial), *psbC* |
| 164,816-166,642 | 1,827 | 38,426-40,358 | 1,933 | *psaB*(partial) |
| 132,403-133,906 | 1,504 | 95,147-96,637 IR | 1,491 | *trnL*, *ndhB*(partial) |
| 54,264-55,607 | 1,344 | 102,191-103,570 IR | 1,380 | *rrn16*(partial) |
| 173,161-174,471 | 1,311 | 44,078-45,435 | 1,358 | *ycf3*(partial) |
| 128,014-129,315 | 1,302 | 109,329-110,994 IR | 1,666 | *trnR*, *trnN* |
| 174,757-176,010 | 1,254 | 24,319-25,572 | 1,254 | *rpoB*(partial) |
| 75,006-76,181 | 1,176 | 86,467-86,574+87,178-88,232 IR | 1,163 | *rpl2*(partial) |
| 172,193-173,129 | 937 | 78,369-79,300 | 932 | *petD*(partial) |
| 168,820-169,540 | 721 | 103,819-104,537 IR | 719 | *trnI*(partial) |
| 95,668-96,211 | 544 | 104,543-105,096 IR | 554 | *trnI*(partial), *trnA*(partial) |
| 36,683-37,178 | 496 | 26,670-27,410 | 741 | *rpoB*(partial) |
| 149,256-149,701 | 446 | 88,209-88,640 IR | 432 | *trnI*, *ycf2*(partial) |
| 94,620-95,040 | 421 | 37,962-38,372 | 411 | *rps14*(partial) |
| 36,033-36,377 | 345 | 25,989-26,356 | 368 | *rpoB*(partial) |
| 161,048-161,354 | 307 | 37,586-37,844 | 259 | *trnG* |
| 112,720-112,957 | 238 | 32,172-32,393 | 222 | *trnY* |
| 94,295-94,511 | 217 | 321-519 | 199 | *psbA*(partial) |
| 148,926-149,134 | 209 | 106,383-106,597^e^ IR | 215 | *rrn23*(partial) |
| 177,545-177,712 | 168 | 105,609-105,775^e^ IR | 167 | *trnA*(partial) |
| 155,209-155,358 | 140 | 32,919-33,060 | 142 | *trnT* |
| Total | 39,563 |  | 38,476 (31,678 excl. repeats/overlaps) | |

The location of the inserts in the mitogenomes is listed for each species and compared to the corresponding region of the plastid genome of *Elodea*. Complete genes or fragments of genes identified in the inserts are also listed.

a) Includes a short overlap

b) Repeated sequence

c-e) Repeated or overlapping sequences

IR = Inverted repeat
