# Supplementary material for: Mitochondrial genome evolution in Alismatales: Size reduction and extensive loss of ribosomal protein genes
Source: PLoS One. 2017 May 17;12(5):e0177606. doi: 10.1371/journal.pone.0177606 (PMC5435185; doi:10.1371/journal.pone.0177606)
Supplement: S6 Table — BLASTN and phylogenetic analyses are done using matrices from Ross et al. [23] including ca. 150 taxa mostly from the Alismatales. (DOCX) [file pone.0177606.s006.docx]

**S6 Table.** **Similarity and phylogenetic relationship of plastid gene fragments located in mitochondrial genomes of *Butomus umbellatus*, *Zostera marina* and *Stratiotes aloides*.**

BLAST and phylogenetic analyses are done using matrices from Ross et al. (2016) including ca. 150 taxa mostly from the Alismatales.

Gene Location Length (bp) Expect Similarity BLAST match Sister group (bootstrap support %)

*atpA* *Stratiotes* 1513 0 99.2% *Stratiotes Stratiotes* (100%)

*atpB* *Z. marina* 1448 0 97.3% *Z. muelleri Z. muelleri* (94%)

*atpE* *Z. marina* 315 5.10e-154 98.9% *Z. muelleri*

*atpF* *Z. marina* 138 7.60e-54 94.9% *Phyllospadix* *Phyllospadix* (48%)

*atpF* *Stratiotes* 555 0 99.6% *Stratiotes* *Stratiotes* + 5 Hydrocharitaceae (19%)

*atpH* *Z. marina* 245 1.04e-120 99.0% *Z. muelleri Z. muelleri* (86%)

*atpH* *Stratiotes* 246 2.02e-123 98.8% *Stratiotes* 6 Hydrocharitaceae (2%)

*atpI* *Stratiotes* 744 0 99.6% *Stratiotes*

*clpP* *Stratiotes* 619 0 91.7% *Stratiotes Stratiotes* (35%)

*infA* *Stratiotes* 244 4.52e-98 93.8% *Stratiotes*

*matK* *Stratiotes* 1488 0 95.8% *Stratiotes Stratiotes* (100%)

*ndhB* *Z. marina* 273 3.09e-118 95,4% *Z. muelleri* *Z. muelleri* + *Phyllospadix* (82%)

*ndhB* *Stratiotes* 536 1.92e-86 85.9% *Potamogeton* RUP+CYM+ZOS+POT^[[1]](#footnote-1)^ (13%)

*ndhF* *Z. marina* 1513 0 93.7% *Phyllospadix* *Z. muelleri* (100%)

*ndhK* *Stratiotes* 596 2.59e-75 70.3% *Illicium Butomus* fragment (100%); both: most angiosperms (78%)

*ndhK* *Butomus* 717 5.29e-123 75.5% *Illicium Stratiotes* fragment (100%); both: most angiosperms (78%)

*petB*ex2 *Stratiotes* 630 0 96.0% *Stratiotes*

*petD* *Butomus* 85 2.45e-31 92.9% *Liriodendron* *Nolina* (13%)

*petD* *Stratiotes* 1229 0 96.1% *Stratiotes* *Stratiotes* + *Hydrocharis* + *Limnobium* (26%)

*petD* *Z. marina* 468 0 97.0% *Z. muelleri* *Z. muelleri* (90%)

*petG* *Stratiotes* 105 6.38e-45 97.0 *Ranalisma* Alismataceae + some Hydrocharitaceae (0%)

*petL* *Stratiotes* 92 6.31e-37 93.5 *Stratiotes*

*psaA* *Stratiotes* 2287 0 92.7% *Stratiotes*

*psaB* *Stratiotes* 2006 0 93.0% *Stratiotes Stratiotes* (100%)

*psaB* *Z. marina* 1682 0 92.9% *Z. muelleri Z. muelleri* (100%)

*psbA* *Stratiotes* 1032 0 99.0% *Stratiotes Stratiotes* (100%)

*psbA* *Z. marina* 154 2.51e-51 92.3% *Z. muelleri Phyllospadix* (4%)

*psbC* *Z. marina* 1298 0 93.7% *Z. muelleri*

*psbD* *Butomus* 160 4.25e-23 73.1% *Nandina* *Enhalus* (1%)

*psbD* *Z. marina* 625 0 90.4% *Z. muelleri* *Z. muelleri* (97%)

*psbE* *Stratiotes* 272 9.31e-116 96.3% *Stratiotes*

*psbF* *Stratiotes* 136 2.25e-33 87.1% *Zomicarpella* Unresolved tree

*psbJ* *Stratiotes* 124 1.71e-53 95.1% *Tetroncium* Unresolved tree

*psbL* *Stratiotes* 119 1.19e-29 87.1 *Stratiotes* + several other alismatids

*rbcL* *Z. marina* 1348 0 94.7% *Z. muelleri Z. muelleri* (100%)

*rpl2*ex1 *Z. marina* 391 0 97.2% *Z. muelleri Z. muelleri* (92%)

*rpl2*ex2 *Z. marina* 95 7.98e-41 94.9% *Z. muelleri*

*rpl14* *Stratiotes* 383 7.14e-133 91.9% *Stratiotes*

*rpl16*ex2 *Stratiotes* 413 0 97.8% *Stratiotes*

*rpl36* *Stratiotes* 244 7.14e-33 89.8% *Stratiotes*

*rpoA* *Stratiotes* 1053 0 90.6% *Stratiotes Stratiotes* (84%)

*rpoB* *Butomus* 157 7.36e-64 93.6% *Pleea* all alismatids (71%)

*rpoB* *Z. marina* 354(a) 1.66e-114 89.8 *Z. muelleri*  *Z. muelleri* (49%)

*rpoB* *Z. marina* 345(b) 1.02e-148 90.5% *Z. muelleri* 3 *Zostera*^[[2]](#footnote-2)^ (79%)

*rpoB* *Z. marina* 1254(c) 0 93.7 *Z. muelleri* 2 *Zostera*^2^ (33%)

*rpoC1*ex2 *Stratiotes* 1351 0 98.2% *Stratiotes*

*rpoC1*ex2 *Z. marina* 761 0 86.9% *Z. muelleri*

*rpoC2* *Stratiotes* 4102 0 97.1% *Stratiotes Stratiotes* (100%)

*rpoC2* *Z. marina* 1492 0 93.6% *Z. muelleri Z. muelleri* (100%)

*rps2* *Stratiotes* 717 0 99.3% *Stratiotes*

*rps3* *Butomus* 63 1.10e-22 98.2% *Xanthosoma* *Musa* (24%)

*rps8* *Stratiotes* 404 0 95.8% *Stratiotes*

*rps11* *Stratiotes* 415 0 95.9% *Stratiotes*

*rps12*ex3 *Stratiotes* 117 5.91e-44 91.4 several alismatids

*rps12*ex2 *Stratiotes* 232 1.17e-117 99.1% *Stratiotes Stratiotes* (56%)

*rps14* *Z. marina* 228 6.73e-105 95.3% *Z. muelleri Z. muelleri* (84%)

*rrn16* *Z. marina* 1289 0 96.7% *Z. muelleri Lepilaena* (2%)

*rrn16* *Butomus* 1492 0 97.7% *Butomus Butomus* (29%)

*rrn16 Stratiotes* 77 1.42e-31 97.4% multiple alismatids + other monocots alismatids + other monocots (27)

*rrn23* *Stratiotes* 2741 0 97.0% *Stratiotes Stratiotes* (33%)

*rrn23* *Z. marina* 2669 0 98.5% *Z. muelleri Z. muelleri* (100%)

*rrn23* *Butomus* 971 0 95.9% *Butomus* 5 Hydrocharitaceae (44%)

*rrn4.5* *Stratiotes* 103 3.65e-51 100.0% *Stratiotes*

*rrn4.5* *Z. marina* 103 3.65e-51 100.0% *Z. muelleri*

*rrn5* *Stratiotes* 113 2.08e-50 93.4% multiple alismatids + other monocots

*rrn5* *Z. marina* 113 2.54e-49 92.6% *Z. muelleri*

*ycf2* *Z. marina* 277(a) 5.31e-110 87.5% *Phyllospadix* not included

*ycf2* *Z. marina* 1904(b) 0 90.2% *Phyllospadix* *Z. muelleri* (99%)

*ycf3* *Stratiotes* 2133 2.21e-106 96.6% *Stratiotes* 8 Hydrocharitaceae (45%)

*ycf3e2* *Z. marina* 217 1.97e-101 95.1% *Z. muelleri Z. muelleri* (96%)

*ycf4* *Stratiotes* 223 4.58e-91 90.9% *Stratiotes*

1. Ruppiaceae, Cymodoceaceae, Zosteraceae, Potamogetonaceae [↑](#footnote-ref-1)
2. The three *Z.marina* fragments branch off in consecutive order [↑](#footnote-ref-2)
