# Supplementary material for: Mitochondrial genome evolution in Alismatales: Size reduction and extensive loss of ribosomal protein genes
Source: PLoS One. 2017 May 17;12(5):e0177606. doi: 10.1371/journal.pone.0177606 (PMC5435185; doi:10.1371/journal.pone.0177606)
Supplement: S7 Table — (DOCX) [file pone.0177606.s007.docx]

**S7 Table. Sequence similarity between the mitochondrial and nuclear genome of *Zostera marina***

| E value | Scaffold no. | Length | % pairwise similarity | MT position | N position | MT annotation | N annotation |
| --- | --- | --- | --- | --- | --- | --- | --- |
| 3.51e-78 | 5 | 258 | 86.0 | 121999-122248 | 665558-665815 | nad5 | Polynucleotide 5'-hydroxyl-kinase grc3 (intron) |
| 3.08e-85 | 7 | 275 | 85.5 | 145890-146139 | 721427-721701 | CP insert | Defective in exine formation protein, putative (intron) |
| 0 | 32 | 520 | 96.3 | 75101-75612 | 575717-576236 | CP insert | Hypothetical protein |
| 1.30e-140 | 35 | 322 | 95.0 | 75544-75865 | 201825-202146 | CP insert | putative insulin-degrading enzyme (intron) |
| 0 | 37 | 1241 | 99.8 | 169600-170840 | 386832-385592 | atp1 | Signal peptide peptidase 2 (intron) |
| 0 | 37 | 775 | 99.6 | 22972-23746 | 385592-386365 | atp1 like fragment | Signal peptide peptidase 2 (intron) |
| 0 | 39 | 756 | 81.6 | 70514-71236 | 192656-193396 |  |  |
| 1.49e-114 | 43 | 327 | 88.7 | 125300-125606 | 296988-296662 | CP insert |  |
| 6.33e-132 | 45 | 322 | 93.2 | 40938-41256 | 819562-819883 | nad6 |  |
| 0 | 54^a^ | 6322 | 98.9 | 97647-103939 | 14245-20542 | nad4 | Hypothetical protein |
| 0 | 54 | 479 | 99.8 | 67938-68416 | 1-479 |  |  |
| 0 | 54 | 3216 | 99.8 | 68480-71695 | 580-3795 | rrn5 trnM | Hypothetical protein |
| 0 | 54 | 7137 | 99.5 | 76567-83703 | 3913-11020 | rps7 ccmFn | Hypothetical protein + putative cytochrome c biosynthesis protein + MT rps7 |
| 0 | 54 | 1583 | 95.7 | 104538-106120 | 20541-22094 | nad4 | Hypothetical protein |
| 4.86e-146 | 54 | 311 | 97.4 | 1-304 | 10758-11068 |  |  |
| 0 | 54 | 965 | 100.0 | 324-1288 | 14245-15209 |  |  |
| 1.81e-151 | 54 | 336 | 96.4 | 13195-13528 | 10689-11024 | nad2 (intron) |  |
| 0 | 54 | 908 | 92.6 | 96782-97627 | 10161-11068 | mttB | Hypothetical protein |
| 1.81e-151 | 54 | 376 | 93.4 | 178395-178766 | 10648-11020 |  |  |
| 0 | 54 | 1026 | 81.0 | 126161-127183 | 17744-16731 |  |  |
| 7.73e-74 | 54 | 269 | 84.4 | 178497-178751 | 10636-10898 |  |  |
| 4.27e-77 | 54 | 288 | 83.2 | 83290-83552 | 10720-11005 |  |  |
| 7.72e-93 | 54 | 365 | 81.6 | 83374-83707 | 10636-10994 |  |  |
| 0 | 79 | 1115 | 90.3 | 75101-76181 | 465573-466645 | CP insert | Hypothetical protein, CP rpl23 |
| 2.21e-74 | 85 | 256 | 85.5 | 74706-74950 | 531531-531279 |  | K(+) efflux antiporter (intron) |
| 2.20e-169 | 104 | 379 | 95.3 | 182299-182670 | 576179-576557 | CP insert | H(+)-transporting two-sector ATPase |
| 2.20e-169 | 104 | 379 | 95.3 | 87236-87607 | 576179-576557 | CP insert | H(+)-transporting two-sector ATPase |
| 0 | 104 | 515 | 90.7 | 143108-143607 | 565438-565951 | CP insert | DNA-directed RNA polymerase |
| 0 | 104 | 833 | 92.0 | 86433-87240 | 575142-575970 | CP insert | H(+)-transporting two-sector ATPase |
| 0 | 104 | 833 | 92.0 | 181496-182303 | 575142-575970 | CP insert | H(+)-transporting two-sector ATPase |
| 0 | 109 | 715 | 84.6 | 168872-169540 | 529665-528986 | CP insert |  |
| 0 | 109 | 715 | 85.0 | 168872-169540 | 526903-526224 | CP insert |  |
| 8.24e-99 | 112 | 286 | 87.4 | 183411-183694 | 282536-282790 | CP insert | Nuclear transcription factor Y subunit B-10 (intron) |
| 8.24e-99 | 112 | 286 | 87.4 | 88348-88631 | 282536-282790 | CP insert | Nuclear transcription factor Y subunit B-10 (intron) |
| 4.27e-77 | 118 | 281 | 80.4 | 111680-111908 | 467454-467176 |  | Alpha-glucan water dikinase (intron) |
| 7.70e-150 | 122 | 309 | 98.7 | 115679-115987 | 467704-468012 | cox2 like fragment |  |
| 7.70e-150 | 122 | 309 | 98.7 | 33197-33504 | 467704-468012 | cox2 like fragment |  |
| 5.20e-95 | 122 | 286 | 87.4 | 93800-94080 | 467736-468012 | cox2 |  |
| 1.94e-138 | 131 | 320 | 95.0 | 40938-41256 | 57055-57374 | nad6 |  |
| 5.18e-171 | 145 | 341 | 99.7 | 34688-35028 | 169868-169528 |  |  |
| 5.18e-171 | 145 | 341 | 99.7 | 117171-117511 | 169868-169528 |  |  |
| 1.07e-84 | 209 | 260 | 87.3 | 121997-122248 | 151093-150834 | nad5 | Polynucleotide 5'-hydroxyl-kinase grc3 (intron) |
| 1.07e-141 | 211 | 319 | 95.6 | 40938-41256 | 176787-176469 | nad6 |  |
| 6.32e-151 | 277 | 321 | 97.8 | 69312-69632 | 149633-149952 |  | Peroxisome biogenesis protein 6 (intron) |
| 4.86e-146 | 278 | 330 | 95.5 | 111032-111361 | 148761-149090 |  |  |
| 2.87e-136 | 359 | 319 | 94.7 | 40938-41256 | 39592-39909 | nad6 |  |
| 6.76e-138 | 370 | 319 | 95.0 | 40938-41256 | 3367-3050 | nad6 |  |
| 1.70e-145 | 446 | 343 | 94.2 | 172805-173145 | 24186-24528 | CP insert |  |
| 2.87e-136 | 456 | 319 | 94.7 | 40938-41256 | 45915-46232 | nad6 |  |
| 2.07e-106 | 552 | 324 | 86.7 | 155210-155533 | 10954-10652 | CP insert | tRNA pseudo |
| 2.21e-112 | 552 | 258 | 95.7 | 112700-112957 | 11592-11335 | CP insert | tRNA-Glu, trnA-Tyr |
| 3.50e-135 | 558 | 319 | 94.4 | 40938-41256 | 51718-51401 | nad6 |  |
| 2.87e-136 | 597 | 319 | 94.7 | 40938-41256 | 20678-20995 | nad6 |  |
| 2.88e-98 | 723 | 319 | 83.7 | 40938-41256 | 30803-30520 | nad6 |  |
| 3.50e-135 | 847 | 319 | 94.4 | 40938-41256 | 1881-1565 | nad6 |  |
| 3.50e-135 | 1153 | 319 | 94.4 | 40938-41256 | 8117-7800 | nad6 |  |
| 2.87e-136 | 5224 | 319 | 94.7 | 40938-41256 | 1391-1708 | nad6 |  |

1. Scaffold 54: Due to assembly gaps in this scaffold matches to positions 67,938-106,120 (38,183 bp) of the mitogenome are broken up in smaller pieces. In the text we refer to this region as the 38 kb sequence.
